# Developmentally programmed histone H3 expression regulates cellular plasticity at the parental-to-early embryo transition

**DOI:** 10.1126/sciadv.adh0411

**Published:** 2023-04-07

**Authors:** Ryan J. Gleason, Yanrui Guo, Christopher S. Semancik, Cindy Ow, Gitanjali Lakshminarayanan, Xin Chen

**Affiliations:** ^1^ Department of Biology, The Johns Hopkins University, Baltimore, MD 21218, USA.; ^2^ Department of Biology, Tufts University, Medford, MA 02155, USA.; ^3^ University of California, San Francisco, San Francisco, CA 94143, USA.; ^4^ Dana-Farber Cancer Institute, Boston, MA 02215 USA.; ^5^ Howard Hughes Medical Institute, Department of Biology, The Johns Hopkins University, 3400 North Charles Street, Baltimore, MD 21218, USA.

## Abstract

During metazoan development, the marked change in developmental potential from the parental germline to the embryo raises an important question regarding how the next life cycle is reset. As the basic unit of chromatin, histones are essential for regulating chromatin structure and function and, accordingly, transcription. However, the genome-wide dynamics of the canonical, replication-coupled (RC) histones during gametogenesis and embryogenesis remain unknown. In this study, we use CRISPR-Cas9–mediated gene editing in *Caenorhabditis elegans* to investigate the expression pattern and role of individual RC histone *H3* genes and compare them to the histone variant, *H3.3*. We report a tightly regulated epigenome landscape change from the germline to embryos that are regulated through differential expression of distinct histone gene clusters. Together, this study reveals that a change from a H3.3- to H3-enriched epigenome during embryogenesis restricts developmental plasticity and uncovers distinct roles for individual *H3* genes in regulating germline chromatin.

## Teaser

Developmentally programmed epigenome changes reset cellular plasticity at the parental-to early embryo transition in *C. elegans*.

## INTRODUCTION

A fundamental and important developmental biology question is how a fertilized egg, upon division and differentiation, becomes a highly organized embryo. On the one hand, gametogenesis represents one of the most marked terminal cellular differentiation pathways. On the other hand, upon fertilization, the totipotency is regained in the zygote (*1*). During these processes, epigenetic information is robustly and extensively established, erased, and reset (*2*–*4*). Subsequently, embryonic cells acquire distinct cell fates through the activation of lineage-specific genes, as well as the repression of nonlineage genes to prevent their ectopic expression. This gene expression program is mediated by chromatin structure and assembly to restrict cellular plasticity upon lineage specification (*5*–*7*).

Histones, the main protein component of chromatin, are essential to the establishment and maintenance of particular chromatin structures, associated with distinct cell fates. The canonical or replication-coupled (RC) histones (i.e., H3, H4, H2A, and H2B) are expressed during S phase and mainly incorporated into the genome during DNA replication. In metazoans, several histone H3 variants have been identified, among which include the replicative and replacement variants, H3 and H3.3, respectively. Histone variants are typically replication-independent (RI), expressed throughout the cell cycle, and regulate a variety of biological processes (*8*–*10*). Unicellular organisms such as yeast only have H3.3-like histones, whereas metazoans have both H3.3- and H3-like histones, suggesting that H3 could be responsible for more specialized roles in metazoan development (*11*, *12*), such as regulating distinct cell fates. Hereinafter, H3 refers to the RC histone, and H3.3 refers to the RI histone variant.

Metazoan RC histones are typically encoded by gene clusters found at multiple chromosomal locations (*13*). For example, the human genome has 14 histone *H3* genes on two chromosomes, while the *Caenorhabditis elegans* genome has 15 histone *H3* genes on four chromosomes (*14*). Moreover, H3 and H3.3 share a 97% similarity among their amino acid sequences and are two of the most conserved proteins among all eukaryotic organisms (*11*, *15*). However, H3 and H3.3 differ in their primary sequences at the 31st and 87th to 90th amino acids (*16*) , exhibit distinct interactions with histone chaperones, and have different genomic distributions (*9*, *17*–*21*). Functionally, H3.3 is often associated with active transcription and enriched with posttranslational modifications (PTMs) such as H3K36me2 and H3K4me3 (*22*–*26*). In contrast, PTMs associated with more repressive chromatin, such as H3K27me2/3 and H3K9me2/3, occur preferentially on H3 (*23*–*25*). Furthermore, recent studies in several organisms have suggested a conserved role for H3.3 during gametogenesis and early embryonic development in mice (*27*–*30*), *Drosophila* (*31*–*33*), and *Xenopus laevis* (*34*). In *C. elegans*, removal of H3.3 is not lethal but reduces fertility and viability in response to stress (*35*).

Because of the dispersed locations and high sequence similarity among the multiple *H3* genomic loci, defining contributions of individual histone *H3* genes has remained a challenge due to the lack of precise genetic tools. Using CRISPR-Cas9–mediated gene editing, we report the endogenous expression patterns and developmental roles of individual histone *H3* genes and compare them to the histone variant *H3.3*, during *C. elegans* gametogenesis and embryogenesis. We show that H3.3 and H3 are enriched in chromatin regions associated with H3K36me2 and H3K27me2/3, respectively. Consistent with these differences in colocalization, knockout alleles of *H3* genes results in a reduction of H3K27me2/3 but an increase in H3K36me2. Last, we provide evidence for the hypothesis that the change from an H3.3- to H3-enriched epigenome during embryogenesis acts as a mechanism to restrict the developmental potential of cells upon lineage specification. Together, our findings reveal a developmentally programmed change in the expression of the multiple histone *H3* genes, which uniquely contribute to the restriction of embryonic plasticity and regulate the chromatin assembly during gametogenesis.

## RESULTS

**Fig. 1.**
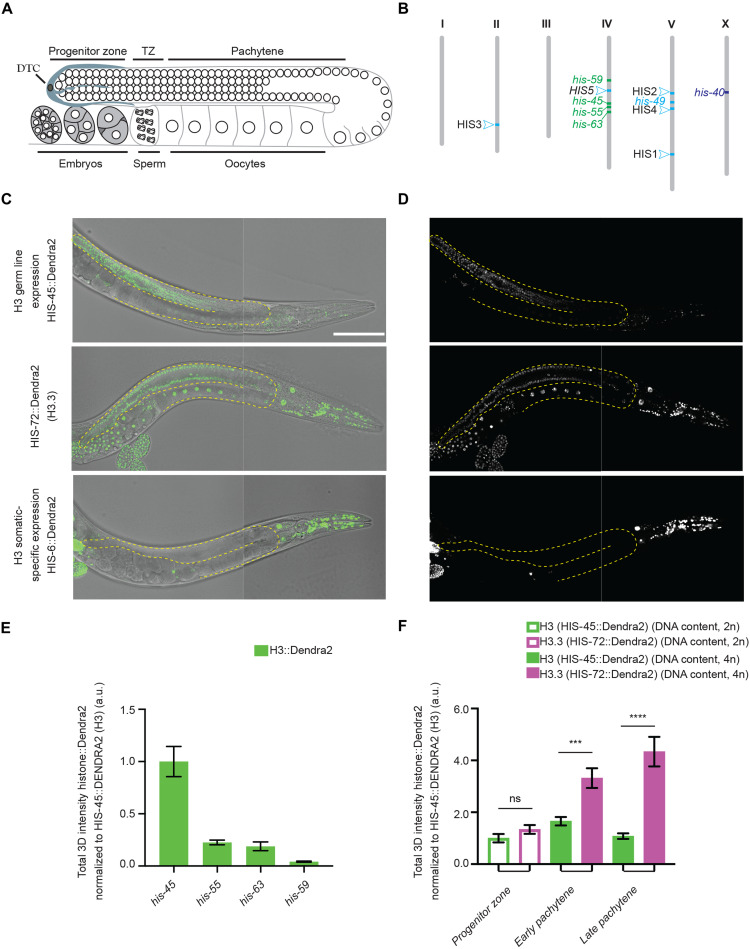
Differential incorporation of the RC histone *H3* gene family. (**A**) Illustration of the *C. elegans* hermaphrodite gonad. DTC, Distal Tip Cell; TZ, transition zone. (**B**) Distribution of the different histone *H3* genes in the genome of the *C. elegans*. Bristol N2 strain is represented using a distinct color code for each class identified, including the ubiquitously expressed class I histone *H3* genes (including both germline and somatic lineages) in green, somatic-specific class II histone *H3* genes in light blue, and *his-40* in dark blue. (**C** and **D**) Representative differential interference contrast (DIC) image and fluorescence micrographs of CRISPR-tagged HIS-45::Dendra2 (H3) (green), the dashed lines outline the gonads. HIS-45::Dendra2 is an example of one of four class I *H3* genes whose expression are detectable in both germline and somatic tissues. CRISPR-tagged HIS-72::Dendra2 (H3.3) shows ubiquitous expression including all somatic tissues and throughout the germline. CRISPR-tagged *H3* gene HIS-6::Dendra2 expression is not detectable in the germline but is detectable throughout the somatic cell lineage, representing the expression pattern of 10 of 15 class II histone *H3* genes. All images from (C) and (D) are taken using the same imaging settings. (**E**) Quantification of all four *H3* genes expressed in the germline. For each condition, a minimum of three biological replicates were performed. The total amount of H3::Dendra2 present per mitotic/progenitor germline nuclei was quantified including *his-45* (*n* = 9), *his-55* (*n* = 15), *his-63* (*n* = 15), and *his-59* (*n* = 18). (**F**) Quantification of the most robust germline-expressed *his-45 H3* gene along the distal-proximal gonadal regions per nuclei: germline stem cell (GSC) region (*n* = 18), early pachytene (*n* = 18), and late pachytene (*n* = 18); and *his-72 H3.3* gene: GSC region (*n* = 21), early pachytene (*n* = 21), and late pachytene (*n* = 21). All quantifications = average ± SE. Unpaired *t* test was used for statistical comparison; ****P≤ 0.0001 and ***P ≤ 0.001. ns, not significant; a.u., arbitrary units. Scale bar, 100 μm.

To investigate the spatiotemporal dynamics of each *H3* gene cluster in the *C. elegans* genome, we used a *dpy-10* co-CRISPR genome engineering strategy to systematically tag endogenous histone genes (*36*, *37*), leading to a series of histone *H3* knock-in lines that produce fusion proteins with different fluorescent tags such as Dendra2, mCherry, and enhanced green fluorescent protein (eGFP) at their C termini (*38*). Using quantitative, live-cell imaging to characterize histone H3 incorporation patterns, we uncovered two major classes of *H3* genes based on their distinct expression patterns during gametogenesis and early embryogenesis. Unexpectedly, only a small subset (4 of 15 *H3* genes, class I) are expressed in both the germline and somatic lineages, which include *his-45*, *his-55*, *his-59*, and *his-63* (Fig. 1, A to D, and fig. S1A). Most *H3* genes (10 of 15, class II) are not detectable in the germline but only in the somatic cell lineages, including members of the HIS1 (*his-2*), HIS2 (*his-6*), HIS3 (*his-9*, *his-13*, *his-25*, and *his-42*), HIS4 (*his-17*, *his-27*, and *his-49*), and HIS5 (*his-32*) histone gene clusters (Fig. 1, C and D, and fig. S1B). Notably, expression patterns among different *H3* genes within the same gene cluster are consistent with each other. Of the 15 histone *H3* genes, *his-40* is unique, considering that it can only be detected in a subset of somatic lineages (fig. S1C and table S1). Consistent with our results, recent RNA profiling assays have revealed two major RC histone gene clusters based on their distinct transcription patterns. The class I histone *H3* genes (*his-45*, *his-55*, *his-59*, and *his-63*) show detectable transcription at the earliest embryonic time points and from the gonad, suggesting their potential roles in intergenerational epigenetic inheritance (*39*). In contrast, the class II histone *H3* genes show a largely zygotic transcription pattern. Here, our results of different H3 protein expression patterns are consistent with their transcription pattern, indicating that their distinct patterns are not subject to differences in their posttranscriptional regulation.

**Fig. 2.**
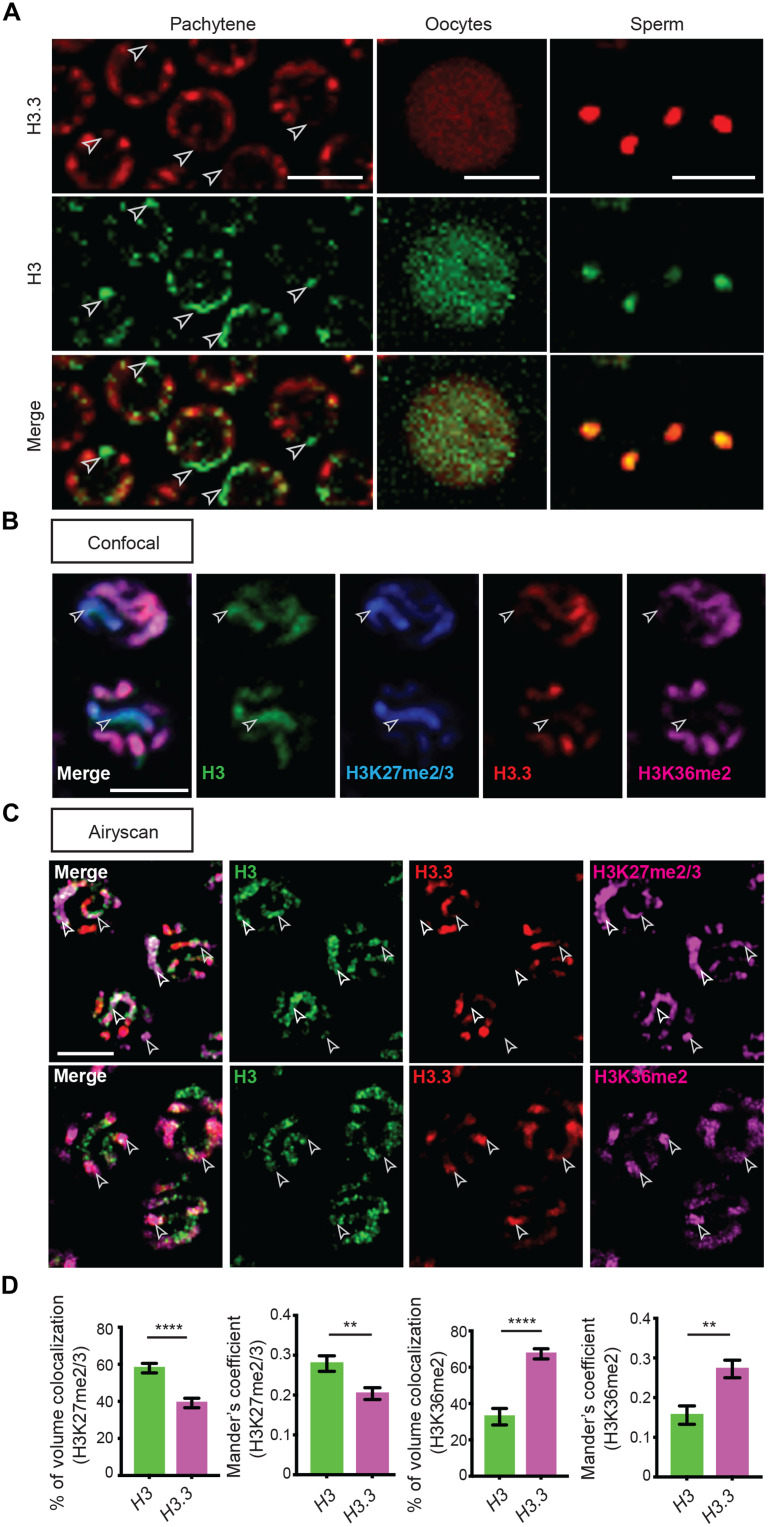
H3 and H3.3 occupy distinct chromatin domains in different staged germ cells. (**A**) High-magnification images of pachytene nuclei, oocyte, and sperm from a young adult hermaphrodite, demonstrating HIS-72::mCherry (H3.3) and HIS-55::GFP (H3). White arrowheads indicate chromatin regions showing presence of H3 but absence of H3.3. (**B**) Immunofluorescence (confocal) of HIS-72::mCherry (red), HIS-55::GFP (green), H3K27me2/3 (blue), and H3K36me2 (magenta) in pachytene nuclei. White arrowheads indicate chromosome X identified by the presence of H3 and H3K27me2/3 but absence of H3.3 and H3K36me2. (**C**) Immunofluorescence (Airyscan) of HIS-72::mCherry (red), HIS-55::Ollas (green), and H3K27me2/3 (magenta; top) and H3K36me2 (magenta; bottom) in pachytene nuclei. White arrowheads indicate distinct chromatin domains marked by the colocalization of H3K27me2/3 or H3K36me2 to the presence of H3 or H3.3, respectively. (**D**) Colocalization analysis of Airyscan immunofluorescent micrographs of H3K27me2/3 or H3K36me2 with H3::Ollas and H3.3::mCherry. All quantifications = average ± SE. Unpaired *t* test; ****P ≤ 0.0001 and **P ≤ 0.01. Scale bars, 5 μm.

In the *C. elegans* gonad, germ cells are organized in a temporally and spatially organized manner along the longitude axis (Fig. 1A). Examination of the four class I *H3* genes revealed that *his-45* is the highest expressed in the germline (Fig. 1E and fig. S1A). To directly compare H3 versus H3.3 quantitatively in a spatiotemporally specific manner, we generated two CRISPR-tagged *H3.3* strains, *H3.3::Dendra2* and *H3.3::mCherry*, respectively (Figs. 1C and 2A). We then used the *H3.3::Dendra2* for quantitative temporal imaging analyses to compare with the *H3::Dendra2* strains. On the other hand, the *H3.3::mCherry* was used with different *H3::eGFP* and *H3::Ollas* strains for their potential distinct localization and chromosomal association. On the basis of these studies, we found that H3 and H3.3 display distinctly dynamic patterns: As germline nuclei move proximally, an approximate fourfold increase in H3.3, encoded by *his-72*, was detected in late pachytene germ cells (Fig. 1F). Consistent with these observations, *his-71*, an alternative *C. elegans* homolog of H3.3, is also preferentially incorporated during late pachytene and is undetectable in the progenitor zone and early meiotic regions (fig. S2). In contrast, H3 is detectable at the distal mitotic region, but the levels decline during germ cell differentiation. In addition, the condensed state of chromosomes during meiotic prophase allows identification of individual chromosomes. Using antibodies against specific histone PTMs, H3K36me2 is enriched on autosomes, while the X chromosome is deficient of H3K36me2 but is primarily associated with H3K27me2/3, consistent with the repressive chromatin status for the X chromosome in *C. elegans* (*40*, *41*). In accordance with previous reports, H3.3 was found to accumulate on the autosomes but was depleted from the X chromosome, based on the anticorrelation of H3.3 with H3K27me2/3 and colocalization of H3.3 with H3K36me2 (*42*–*44*). These differences were consistently visible using conventional confocal microscopy (Fig. 2, A and B) but is more obvious using high spatial resolution Airyscan microscopy (Fig. 2C and movies S1 and S2) (*45*). Contrastingly, germline-expressed H3 was enriched in H3.3-depleted regions, including the X chromosome, marked by the colocalization of H3 with H3K27me2/3 but anticorrelation of H3 with H3K36me2, using both confocal (Fig. 2B) and Airyscan (Fig. 2C and movies S1 and S2). Quantification of these images demonstrates significantly higher degree of colocalization of H3 than that of H3.3 with H3K27me2/3 and significantly higher degree of colocalization of H3.3 than that of H3 with H3K36me2, using two different quantification methods (Fig. 2D and see Materials and Methods). Furthermore, we found that both H3.3 and H3 are retained in mature sperm and oocyte chromatin (Fig. 2A), indicating that they may regulate the transition from germline to zygote.

**Fig. 3.**
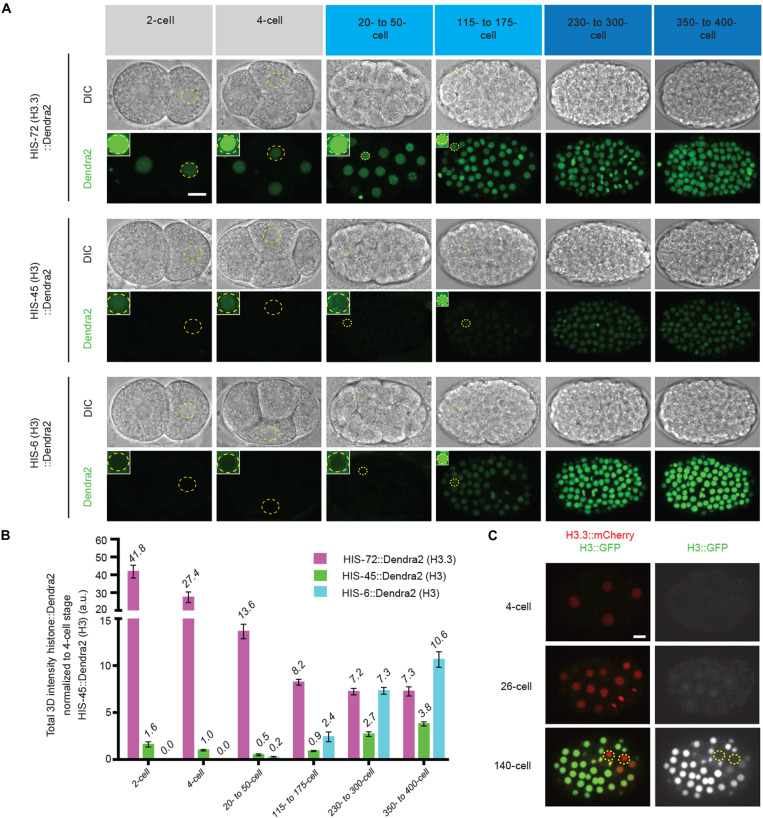
The early embryonic epigenomic landscape is developmentally programmed to switch from histone variant H3.3-enriched to histone H3-enriched upon gastrulation. (**A**) Representative images of embryos expressing H3.3-encoding *his-72::Dendra2*, germline-expressed, H3-encoding *his-45::Dendra2*, and somatic H3-encoding *his-6::Dendra2* throughout the designated stages of embryogenesis including pregastrula (two- and four-cell; gray), earliest stages of gastrulation (21- to 50-cell and 115- to 175-cell; light blue), and after the onset of gastrulation (230- to 400-cell; dark blue), DIC (top), and Dendra2 (bottom). All images from (A) are taken using the same imaging settings, insets demonstrate that *his-72* (H3.3) is readily detectable, whereas *his-45* (H3) is barely detectable by increasing the brightness; however, *his-6* (H3) is undetectable even with enhanced brightness but only becomes detectable at the onset of gastrulation (at the 26-cell stage). (**B**) Quantification of data sets from (A). For each embryonic stage, the total amount of Dendra2 representing either H3 or H3.3 per nuclei were quantified. All quantifications = average ± SE. (**C**) Snapshots from time-lapse movies of embryos expressing HIS-72::mCherry (H3.3) and HIS-55::GFP (H3) (see movie S3). The dashed circles outline the primordial germ cells. Scale bars, 10 μm.

**Fig. 4.**
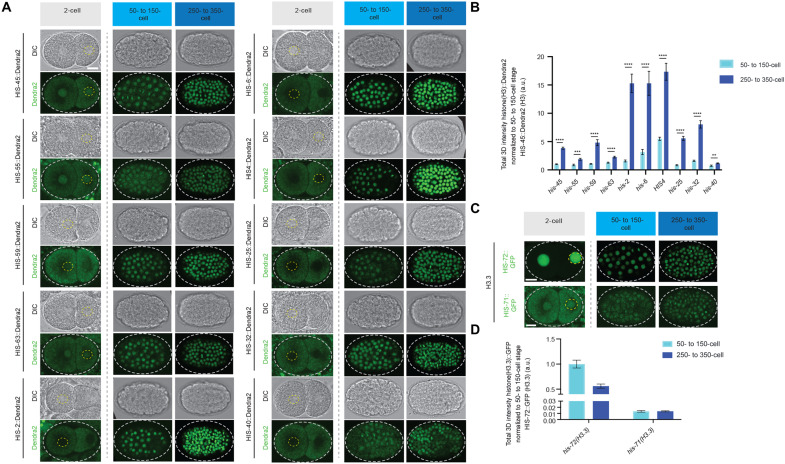
Expression patterns of all histone H3 gene clusters and H3.3 genes during early embryonic development. (**A**) Representative fluorescent micrographs of class I RC histone H3 isotypes (*his-45*, *his-55*, *his-63*, and *his-59*) and for one member from each of the five histone gene clusters including HIS1 (*his-2*), HIS2 (*his-6*), HIS3 (*his-25*), HIS4 (*his-17*, *his-27*, and *his-49*) (see Materials and Methods for details), and HIS5 (*his-32*). The yellow and gray dashed lines outline the nuclei and embryo, respectively. Class I histone *H3* genes are detectable in the early stages of embryogenesis, represented by the two-cell stage. Class II histone *H3* genes are undetectable at the early stages of embryogenesis before the onset of gastrulation. All two-cell images from (A) were taken using the same imaging settings, DIC (top), and Dendra2 (bottom). All class I and class II histone *H3* genes are detectable during gastrulation (50- to150-cell stage) and increase in expression during embryogenesis, represented by the expression captured at the 250- to 350-cell stage, DIC (top), and Dendra2 (bottom). All images representing 50- to 150- and 250- to 350-cell stages were taken using the same image settings. (**B**) Quantification of data sets from (A). For each embryonic stage, the total amount of the Dendra2 signal representing each *H3* gene per nuclei was quantified. (**C**) Representative fluorescent micrographs of the two *H3.3* genes (*his-72* and *his-71*). Two-cell images from (C) were not taken using the same imaging settings. Unlike histone H3, the *H3.3* genes, *his-72* and *his-71*, do not increase significantly during early embryogenesis. All images representing 50- to 150- and 250- to 350-cell stages were taken using the same image settings. (**D**) Quantification of data sets from (C). For each embryonic stage, the total amount of GFP representing both H3.3 genes per nuclei was quantified. All quantifications = average ± SE. Unpaired *t* test; ****P < 10^−4^, ***P < 10^−3^, and **P < 10^−2^. Scale bars, 10 μm.

In *C. elegans*, embryos initiate zygotic transcription in the blastomeres beginning at the 4-cell stage, while the onset of gastrulation begins at the 26-cell stage (*46*–*48*). We observed a low level of H3 (represented by a class I *his-45* gene and a class II *his-6* gene) but a high level of H3.3 (represented by the *his-72* gene) throughout the rapid cell cycles of early embryogenesis (Fig. 3A). Further analyses of all other class I histone H3 genes (*his-55*, *his-59*, and *his-63*) and one member from each of the five histone gene clusters, including HIS1 (*his-2*), HIS2 (*his-6*), HIS3 (*his-25*), and HIS4 (*his-17*, *his-27*, and *his-49*), showed consistent patterns with *his-45* and *his-6* genes (Fig. 4A). However, when gastrulation initiates, a marked increase in all 15 histone *H3* genes was detected (Figs. 3, A and B, and 4, A and B). Consistent with the above results (Fig. 1), the expression timing of the *H3* genes coincide with each other within the same class (i.e., class I versus class II), suggesting that they are developmentally coordinated for coexpression. Using *his-45* (class I) and *his-6* (class II) to represent the two classes of *H3* genes, the *his-45* was detectable throughout embryogenesis, including the earliest cell divisions, and increased gradually upon gastrulation (Fig. 3, A and B). Expression of the soma-specific *his-6* gene was not detectable until the onset of gastrulation and increased rapidly over the subsequent cell divisions (Fig. 3, A and B). Most highly expressed class II *H3* genes at the late-stage 250- to 350-cell embryos are on chromosome Ch V, such as HIS2 (*his-6*), HIS4 (*his-17*, *his-27*, and *his-49*), and HIS1 (*his-2*), while all four germline-expressed class I *H3* genes (*his-45*, *his-55*, *his-59*, and *his-63*) are on a different chromosome Ch IV (Figs. 3, A and B, and 4, A and B), suggesting potential cis-regulatory mechanisms for the coexpression patterns of different classes of *H3* genes. On the other hand, neither *his-71* nor *his-72* gene that encodes H3.3 showed significantly increased expression during gastrulation (Fig. 4, C and D). Quantifying expression of these two H3 classes and H3.3 genes highlights the global change in the epigenome, from an H3.3-enriched epigenetic landscape in late germline and early embryos to an H3-enriched epigenome in late embryos (Figs. 3, A to C, and 4, A to D). Furthermore, this change is specific to the somatic lineage cells, as live-cell imaging revealed that the P lineage for primordial germ cells does not display such an increased H3 incorporation throughout embryogenesis but remains enriched with H3.3 (Fig. 3C and movie S3).

We next asked whether the germline expressed class I *H3* genes are required for normal gametogenesis and fertility. To address this question, we generated deletion alleles in two of the lowly expressed class I *H3* genes, *his-59* and *his-55*, using CRISPR-Cas9–mediated genome editing. Removal of *his-59* and *his-55* is already sufficient to result in detectable phenotypes. We observed a variable penetrance of shrunken and collapsed germlines that exhibit a reduction in germ cell number. To understand whether this germ cell loss is due to a reduction of mitotic or/and meiotic cells, we used a reporter that specifically labels the mitotic cells by GFP (*49*), while all germ cells are labeled by mCherry. Therefore, mitotic cells are GFP^+^mCherry^+^, while meiotic cells are GFP^−^mCherry^+^. Comparing wild-type with the *his-59*; *his-55* mutant gonads, the size of the progenitor mitotic zones was comparable, whereas the meiotic regions were significantly reduced in the double *his-59*; *his-55* mutant gonads (fig. S3, A and B). Consistent with the high degree of colocalization of H3 with H3K27me2/3 (Fig. 2C-D), in the germline from *his-59*; *his-55* double mutants, there is a significant decrease in the H3K27me2/3 immunostaining signals (fig. S3, C and D). Contrastingly, the H3K36me2 enriched on H3.3 (Fig. 2, C and D) displayed increased signals in the *his-59*; *his-55* double mutant germ cells (fig. S3, C and D). In addition, *his-59*; *his-55* double mutants displayed a mild but statistically significant reduction in brood sizes (fig. S3E). To assess whether the germ cell loss and brood size reduction were due to increased cell death, we used a CED-1::GFP reporter to visualize apoptotic germ cells in adults (*50*). Increased germ cell apoptosis was already detectable in the *his-59* single mutant, and such a phenotype was enhanced in the *his-55*; *his-59* double mutant (fig. S3, F and G). These data indicate that compromising H3 levels in the germline by removing two class I *H3* genes is sufficient to cause a reduction in germ cell nuclei, an increase in apoptosis, and a moderate reduction in brood size. In contrast, previous studies demonstrate that H3.3 is dispensable, as knockout of the *H3.3* genes did not lead to detectable germline defects, using a strain that lacks the two H3.3 homologs—*his-71* and *his-72*—and the H3.3-like genes—*his-69*, *his-70*, and *his-74* (*35*). The impact of histone H3 on germ cell loss and apoptosis is phenotypically similar to the loss of chromatin repressors such as the polycomb repressive complex 2 (*mes-2*, *mes-3*, and *mes-6*) and the H3K9 methyltransferase, *met-2*, respectively (*51*, *52*).

**Fig. 5.**
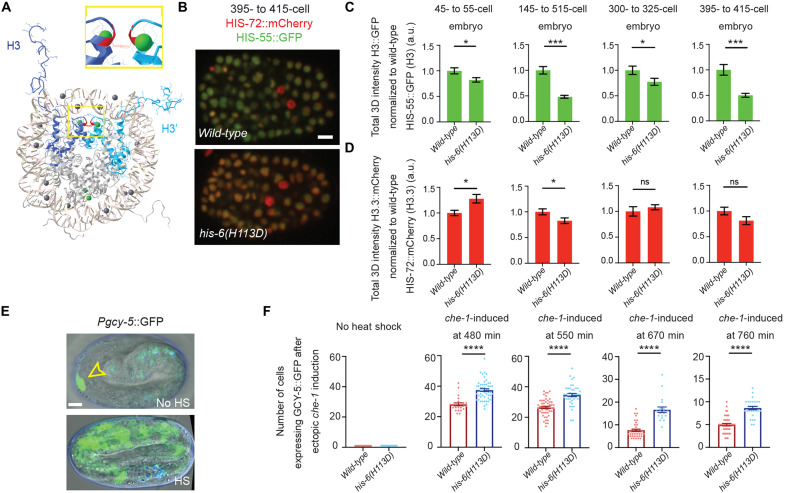
A dominant-negative mutation in the somatic-specific histone *H3* gene, *his-6(H113D)*, destabilizes the histone tetramer (H3-H4)2, leading to reduced H3 incorporation and extended embryonic plasticity. (**A**) Structure of histone H3 in the histone octamer, drawn with Cn3D (www.ncbi.nlm.nih.gov/Structure/CN3D/cn3d.shtml). The two H3 in the octamer (H3 and H3′) are colored in dark blue (H3) and light blue (H3′). The interface of H3 and H3′ maintains the (H3:H4)2 tetramer, for which the H113 (Red) → D mutation in the somatic H3-encoding *his-6* gene has a dominant-negative effect by destabilizing the (H3:H4)2 tetramer specifically in somatic cells. A zoomed in view is displayed in the inset. (**B**) Representative images of embryos expressing H3.3::mCherry by *his-72::mCherry* and H3::GFP by *his-55::GFP* at the 395- to 415-cell stage to compare the H3::GFP levels in wild-type versus *his-6(kog10)*(*H113D*) mutant embryos. (**C** and **D**) Quantification of *his-72::mCherry (H3.3)* and *his-55::GFP (H3)* embryos at designated stages in wild-type vs. *his-6(kog10)*(*H113D*) embryos. (**E**) Embryonic plasticity assay: The top panel displays an embryo without heat-shock induction of *che-1* expression, only the ASER neuron is labeled with bright *gcy-5::GFP* expression (yellow arrowhead) (signals that overlap with the blue channel are considered autofluorescence). The bottom panel is an example of heat-shock induced embryonic *che-1* expression at 550 min into embryogenesis, *gcy-*5 expression is broadly activated. (**F**) Quantification of the number of cells per embryo expressing *gcy-5::GFP* in response to *che-1* induction at different staged embryos. At each tested stage, embryos carrying the dominant negative mutation of *his-6(kog10)*(*H113D*) display more *gcy-5::GFP*-expressing cells per embryo challenged during the same time points including 480, 550, 670, and 760 min during embryogenesis, *his-6(H113D)* embryos resulted in a 38, 33, 118, and 72% increase in cells that are converted, respectively. Scale bars, 5 μm. All quantifications = average ± SE. Unpaired *t* test; ****P ≤ 0.0001, ***P ≤ 0.001, and *P ≤0.05.

Before gastrulation, embryonic blastomeres are characterized by decondensed chromatin and wide cellular differentiation capacity, termed as pluripotent (*6*, *53*, *54*). As embryos transit through gastrulation, they acquire distinct cell fates, accumulate heterochromatin, and restrict the developmental plasticity (*6*, *55*, *56*). To determine whether the increased incorporation of H3 during gastrulation is a mechanism for restricting embryonic plasticity, we generated a mutated form of H3 containing a single substitution of histidine (His or H) to aspartic acid (Asp or D) at the 113th position (H113D). The C-terminal H113 of H3 is a key residue that functions at the interface between the two H3-H4 dimers (H3 and H3′ in Fig. 5A). By changing the positively charged H to the negatively charged D, the H113D mutant is thought to markedly destabilize the H3:H3′ interface, preventing the complex formation between an H3-H4 dimer and the histone chaperone CAF-1. This mutant has been reported to have a dominant negative inhibition on CAF-1–mediated nucleosome deposition in vitro and in vivo (*57*, *58*). To avoid detrimental developmental defects, we generated this mutation at the *his-6* locus, a somatic-specific class II histone *H3* gene, which limits the expression of the H113D restrictively in the somatic lineage and specifically at the onset of gastrulation. The *his-6(H113D)* mutant worms were viable, with no apparent embryonic abnormalities or change in the timing of embryogenesis. We then examined whether H113D acts dominant negatively to reduce the deposition of wild-type H3. When crossing the *his-6(H113D)* mutant into a reporter strain carrying both *his-55::GFP* (H3) and *his-72::mCherry* (H3.3), the *his-6(H113D)* resulted in a significantly decreased H3 incorporation during embryogenesis, when comparing to the wild-type strain at multiple time points in early embryonic development (Fig. 5, B and C). By contrast, H3.3 incorporation showed either inconsistent (first and second histograms) or insignificant (third and fourth histograms) changes at the comparable embryonic developmental time points (Fig. 5D).

The molecular processes underlying declined cellular plasticity during embryogenesis have been previously investigated (*6*, *54*, *59*). For example, all somatic lineages can be converted into other somatic cell types, depending on the ectopic expression of master cell fate defining transcription factors (*60*–*62*). However, this reprogramming flexibility is gradually lost in cells from embryos after initiating gastrulation (*6*, *56*, *60*, *63*–*65*). It has been shown that ectopic expression of the transcription factor *che-1/*Zn-finger, which is normally expressed solely in the bilaterally symmetric ASE neuron pair (ASEL and ASER), is sufficient to induce the expression of a neuronal cell fate reporter (*gcy-5::GFP*) and aberrant adoption of neuronal cell fates in early embryonic blastomeres (*56*, *66*–*69*). However, the ability of blastomeres to respond to ectopic expression of *che-1* is progressively lost during gastrulation (*56*). To determine whether the developmentally programmed increase in H3 deposition during gastrulation restricts the embryonic plasticity, we challenged embryos to adopt alternative cell fates at progressively later developmental time points. Without ectopic expression of *che-1*, the neuronal cell fate reporter, *gcy-5::GFP*, is expressed solely in the ASER neuron in late-stage embryos (Fig. 5, E and F). In this assay, non-ASE cells that actively express *gcy-5::GFP* in response to *che-1* ectopic expression are considered plastic. When *che-1* is induced during early embryonic stages (480 and 550 min) in a wild-type background, *gcy-5::GFP* is broadly activated (Fig. 5F). Consistent with the progressive loss of plasticity, wild-type embryos that are challenged later in embryogenesis (670 and 760 min) demonstrate a reduced ability to respond to ectopically expressed *che-1* by turning on *gcy-5::GFP* (Fig. 5F). In contrast, in *his-6(H113D)* embryos, the number of cells that respond to *che-1* overexpression exhibited a significant increase in neuronal cell fate induction and *gcy-5::GFP* expression, when compared to wild-type controls throughout embryogenesis (Fig. 5F). Together, these results demonstrate that histone H3 incorporation during gastrulation progressively restricts embryonic plasticity and that reduced H3 incorporation is sufficient to extend the window of embryonic plasticity.

**Fig. 6.**
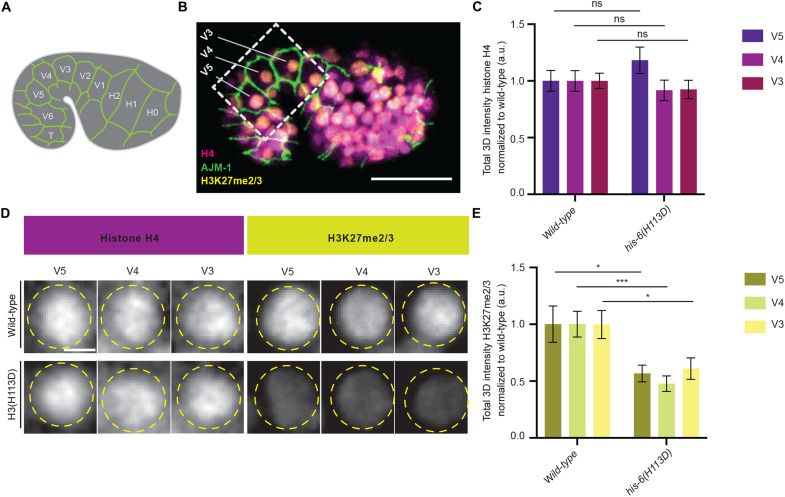
The dominant-negative allele of H3, *his-6(H113D)*, does not significantly change the total nucleosome levels but does lead to reduced levels of H3K27me2/3. (**A**) Illustration of *C. elegans* comma stage embryos expressing AJM-1::GFP reporter in epithelial cells to label 10 embryonic seam cells (H0-T). (**B**) Representative fluorescent micrograph of Histone H4 (magenta) and H3K27me2/3 (yellow) levels in a wild-type embryo expressing AJM-1::GFP at the comma stage of embryogenesis. (**C**) Quantitation of Histone H4 levels per nucleus in specific epithelial cell types including V5, V4, and V3 in wild-type versus *his-6(H113D)* at the comma stage of embryogenesis. The total amount of histone H4 present per nuclei was quantified in V5 {*n* = 21 (wild-type) and 19 [*his-6*(H113D)]}, V4 {*n* = 17 (wild-type) and 18 [*his-6*(H113D)]}, and V3 {*n* = 21 (wild-type) and 19 [*his-6*(H113D)]}. (**D**) Representative Immunofluorescence micrographs of histone H4 and H3K27me2/3 in V5, V4, and V3 nuclei at the comma stage of embryogenesis. (**E**) Quantitation of H3K27me2/3 levels per nucleus in specific epithelial cell types including V5, V4, and V3 in wild-type versus *his-6(H113D)* at the comma stage of embryogenesis. The total amount of H3K27me2/3 present per nuclei was quantified in V5 {*n* = 12 (wild-type) and 13 [*his-6*(H113D)]} (*P = 0.0185), V4 {*n* = 8 (wild-type) and 12 [*his-6*(H113D)]} (***P = 0.0005), and V3 {*n* = 11 (wild-type) and 12 [*his-6*(H113D)]} (*P = 0.020). All quantifications = average ± SE. Unpaired *t* test was used for statistical comparison; ***P ≤ 0.001 and *P ≤ 0.05. Scale bars, 20 μm (B) and 2 μm (D).

Unlike histone H3, which has diverse variant forms, histone H4 is invariant and encoded by only one isotype in fungi, plants, and animals (*15*). Thus, every histone octamer contains two identical histone H4 proteins. Therefore, measuring histone H4 levels per nuclei provides a relative metric to compare total nucleosome levels between samples. To examine whether the increase in embryonic plasticity observed in *his-6(H113D)* animals is a result of a change in the total nucleosome levels, we compared histone H4 levels in *his-6(H113D)* mutants to wild-type embryos (Fig. 6). Because our cell fate challenge assay indicates that *his-6(H113D)* leads to a gain of plasticity by the 480-min early embryonic time point, we chose to focus on embryos between the comma and 1.5-fold stage, which occur between 430 and 490 min into embryogenesis. Furthermore, we chose to focus on the epithelial, seam cell lineage for the following reasons: First, they are readily and precisely identifiable at the comma stage and bounded by AJM-1::GFP, confirming their seam identity (Fig. 6, A and B). Second, while some lineages have begun to terminally differentiate, the seam cells we selected, including V3, V4, and V5, will undergo further divisions. Third, V3 to V5 are located at the periphery of the embryo that is optimal for immunostaining experiment and image analysis. We found that total histone H4 levels did not change significantly between wild-type and *his-6(H113D)* mutants (Fig. 6, C and D). Previous work in human cell lines indicated that when the canonical H3 deposition is impaired, alternative histone chaperone pathways including the histone regulator A (HIRA) complex, which mediates RI nucleosome assembly and H3.3 incorporation, can provide a nucleosome gap-filling strategy at any region where non-nucleosomal/naked DNA is accessible (*70*). Therefore, our finding reflects the potential for restoration of total nucleosome levels using any available H3 variants and/or histone chaperone pathways to maintain chromatin stability when CAF-1–mediated nucleosome assembly is impaired in *his-6(H113D)* mutants. Consistently, when wild-type and *his-6(H113D)* mutant embryos were costained to compare H3K27me2/3 levels, we found that *his-6(H113D)* mutant embryos have a significant decrease in H3K27me2/3 levels by the comma/1.5-fold stage compared to the wild-type control strain (Fig. 6, D and E). This indicates that reducing the CAF-1–mediated deposition of canonical H3 affects the establishment and/or maintenance of H3K27me2/3 during embryogenesis, consistent with previous studies in mammals and plants (*71*, *72*). In summary, we conclude that the extension of embryonic plasticity detected in *his-6(H113D)* mutants is likely due to a change in the chromatin landscape, including H3K27me2/3 levels, but not due to a change in the total nucleosome levels.

## DISCUSSION

In metazoans, histone genes are clustered together. For example, in the human genome, a large cluster on chromosome 6 and two small clusters on chromosome 1 contain all the RC histone genes. While in the mouse, a large cluster on chromosome 13 and two small clusters on chromosome 3 contain all RC histone genes (*14*, *73*). In *C. elegans*, several clusters of histone genes are dispersed on four distinct chromosomes (Fig. 1B). Here, our results demonstrate that the expression of each histone cluster is developmentally regulated for tightly coordinated expression, which is important for embryonic development and germline fitness. Results from other metazoan embryos, such as sea urchin, suggest a potentially conserved regulation of distinct histone gene clusters during embryogenesis (*74*, *75*). For example, sea urchins have two sets of RC histone genes. From the oocyte stage until gastrulation, histone H3 is expressed from a subset of histone genes designated as the “early” subtype (*75*). Upon the onset of gastrulation, during the mesenchyme-blastula stage, a separate set of histone genes designated as the “late” subtypes are synthesized throughout the differentiated embryo and throughout the remaining life cycle (*76*). Therefore, our results start to shed light on the functional roles of selective histone cluster expression during development. Given the conserved clustering features of histone genes in different metazoan species, it will be important to investigate whether such a programmed histone gene expression is a common feature and what cue(s) may coordinate the developmental timing and tissue specificity of individual histone genes.

Despite their widely divergent morphologies, invertebrates and vertebrates go through largely similar early embryonic stages. For example, it has been shown that the de novo establishment of heterochromatin domains during early embryogenesis is conserved from *Drosophila*, *C. elegans*, and zebrafish to mammals (*55*, *77*–*79*). Heterochromatin is known to be a potent epigenetic barrier against cellular reprogramming in *C. elegans* and in mammals (*56*, *80*–*82*). In *C. elegans*, the timing of heterochromatin formation during embryogenesis has been tracked using transmission electron microscopy and immunostaining with antibodies against heterochromatin-enriched PTMs (*55*). These studies have provided evidence that heterochromatin domains are established during gastrulation, coinciding with the marked increase in *H3* gene expression and H3 incorporation identified in this work, in particular, the class II *H3* genes. Here, beyond identifying a global change from a H3.3-enriched to a H3-enriched epigenome and a developmental role for this change in regulating embryonic plasticity, we further provide a mechanistic insight into the specific impact of reducing H3 incorporation during embryogenesis. We reveal that during embryogenesis, reduction of H3 does not change the overall nucleosome levels but is sufficient to result in a reduction in H3K27me2/3, a repressive histone PTM. Consistent with our results, extension of embryonic plasticity has also been identified in animals deficient in histone PTMs associated with heterochromatin, such as H3K27 and H3K9 methylation (*6*, *56*). Furthermore, recent studies in fission yeast have demonstrated that a critical density threshold of an H3K9me3 is required to promote epigenetic inheritance of heterochromatin (*83*). Therefore, this mechanism of increased H3 incorporation during gastrulation may be especially critical for establishing and/or maintaining repressive chromatin domains that, in turn, mediate embryonic plasticity and cell fate maintenance. Additional factors may also work together with the dynamic up-regulation of chromatin-associated H3 to coregulate heterochromatin formation. Recent studies in *C. elegans* have identified that MET-2, an H3K9 methyltransferase, translocates from the cytosol to the nucleus during gastrulation (*55*). Together, our findings can be viewed from the perspective of a multistep process of development that contributes to the dynamic epigenetic changes, which occur during germ cell development, to the establishment of early embryonic chromatin, and ultimately to a differentiated somatic cell type.

The hypothesis that the change from an H3.3- to an H3-enriched epigenome may influence the distribution of H3K27me2/3 across the genome during embryogenesis can be tested by performing cell type–specific chromatin immunoprecipitation sequencing experiments in *C. elegans* along with comparative analysis of H3 and H3.3 incorporation before and after gastrulation. Mouse early embryos have recently been found to transit from an H3.3-enriched epigenetic landscape where H3.3 is distributed evenly across the genome to a more canonical pattern, in which H3.3 becomes enriched at active chromatin (*84*). Further analysis will be required to investigate whether a conserved expression change from H3.3 to H3 underlies this epigenome change during mammalian embryogenesis.

Histone PTMs have also been found to transit from both maternal and paternal chromatin to the zygote in *C. elegans* and in mammals (*85*–*87*). For example, H3K27me3, which is generated by the polycomb repressive complex 2, can be inherited both maternally and paternally (*86*). Therefore, histone PTMs established during oogenesis and spermatogenesis transmit an epigenetic memory from gametes to the zygote (*86*). Future investigations will be needed to determine whether unique histone PTMs are inherited preferentially on H3 or H3.3 and where H3 and H3.3 are distributed throughout the parental and early embryonic genomes in *C. elegans*.

## MATERIALS AND METHODS

### *C. elegans* strains

*C. elegans* were maintained on nematode growth medium agar plates using *Escherichia coli* OP50 as a food source and cultured according to standard methods (*88*). For heat-shock experiments, worms were grown at 15°C, heat-shocked at 32°C for 30 min, left at 20°C overnight, and scored about 24 hours later. Strains used and generated for this study are listed in table S2.

### CRISPR-Cas9–generated alleles

CRISPR engineering was generated by microinjecting Cas9–guide RNA (gRNA) ribonucleoprotein complexes into hermaphrodite gonads as described previously (*36*). Unique gRNA sequences were selected using the off-target predictions CRISPR design tool at https://crispr.mit.edu. For large edits, such as fluorescent protein tag sequences, we generated double-stranded DNA repair templates by amplifying eGFP, mCherry, or Dendra2 by polymerase chain reaction using specific oligos containing homology arms of approximately 35 base pairs (bp). For histones H3 and H3.3, the fluorescent protein sequence for Dendra2, GFP, or mCherry was inserted into the C terminus of the protein just before the stop codon. Tagging at the C terminus was based on published H3.3 and H3 protein fusion analysis (*89*, *90*). For single-nucleotide modifications or deletions, we used single-stranded oligonucleotides containing homology arms of approximately 35 bp as repair templates ordered from Integrated DNA Technologies, as standard 4 nM ultramer oligos. Silent mutations were included where necessary in the repair templates to modify either the PAM sequence or the gRNA seed region to prevent Cas9 from cleaving the repair template. (See table S3 for molecular details of gene editing reagents).

### Immunocytochemistry

Germlines were dissected from 24-hour post-L4 adult hermaphrodites in egg buffer [25 mM Hepes (pH 7.4), 118 mM NaCl, 48 mM KCl, 2 mM EDTA, 5 mM EGTA, 0.1% Tween 20, and 15 mM NaN_3_] and fixed in 1% formaldehyde. Following fixation, samples were covered with a coverslip to ensure attachment to the slide surface and flash-frozen on aluminum blocks chilled on dry ice. The samples were then fixed for 1 min in 100% methanol (−20°C) and rehydrated with phosphate-buffered saline (PBS) containing 0.1% Tween 20 (PBST). Samples were then blocked for at least 30 min in PBS containing 0.1% bovine serum albumin (BSA) and PBST. Primary antibodies were diluted in PBST and BSA at the following concentrations: mouse anti-H3K27me2/3 diluted 1:5000 (39535, Active Motif, RRID:AB_2793246), chicken anti-GFP diluted 1:250 (ab13970, Abcam, RRID:AB_300798), rat anti-Ollas diluted 1:500 (NPB1-06713, Novus, RRID:AB_1625979), histone H4 (ab31830, Abcam RRID:AB_1209246), and rabbit anti-H3K36me2 diluted 1:50 (ab9049, Abcam, RRID:AB_1280939). All primary antibody incubations were overnight at 4°C. Slides were washed three times with PBST for 10 min each and incubated with secondary antibodies for 2 hours. Secondary antibodies were the Alexa Fluor–conjugated series (1:1000; Molecular Probes). Confocal images for immunostained fixed samples were taken using a Zeiss LSM 780 confocal and Zeiss 800 Airyscan microscopes with 63× objectives and processed using Imaris (Bitplane), Fiji, and Adobe Illustrator software.

### Microscopy and image analysis

For imaging experiments, many strains were surveyed under the microscope through the eye piece, and general trends were noted for each strain generated. Then, a random subset of selected animals was imaged and analyzed more deeply with quantitation. Live worms and embryos were mounted on 2% (w/v) agarose pads. Live images were collected from 24-hour post-L4 adult hermaphrodites in M9 buffer and tetramisole (100 mM) using a Zeiss 780 laser scanning confocal microscope. To stage embryos, two-cell embryos were selected and imaged using a Zeiss 780 laser scanning confocal or a Zeiss spinning disk CSU-X1M for time-lapse imaging. Quantification of images was performed using the open-source Fiji software (*91*). Within any set of comparable images, the image capture and scaling conditions are identical. For each reporter, three randomly selected nuclear regions per animal were analyzed. To quantify the total amount of tagged histone protein per nuclei, we conducted a 3D quantification by measuring the fluorescence signal in each plane from the Z stack. Specifically, raw images as 2D Z stacks were saved as 16-bit TIF images, and the sum of the gray values of pixels in the image (“RawIntDen”) was determined using Fiji. A circle was drawn to include all fluorescence signal (marked by Dendra2, GFP, or mCherry), and an identical circle was drawn outside the sample area as the background. The gray values of the fluorescence signal pixels for each Z stack were calculated by subtracting the gray values of the background signal from the gray values of the raw signal pixels. The total amount of fluorescence signal in the nuclei was then calculated by adding the gray values from all Z stacks. All quantifications of tagged histone *H3* and *H3.3* samples were done using this method.

To quantify the colocalization of tagged histone proteins, H3::Ollas and H3.3::mCherry, with specific PTMs (H3K27me2/3 and H3K36me2) per nuclei, we used the Imaris intensity-based colocalization analysis. After building a colocalization channel, the colocalization statistics for percent of volume A (designated using the PTM channel) above threshold colocalized with volume B (the tagged histone channel) and Mander’s coefficient were quantified per 3D nuclei. Within each dataset of comparable samples, the image capture settings were maintained as identical. For each sample, three randomly selected germline nuclei were analyzed. For each condition, at least three biological replicates were performed for H3K27me2/3 and for H3K36me2.

Quantification of immunofluorescent intensity of histone PTMs was performed using the Imaris (Bitplane) software. H3K27me2/3- and H3K36me2-positive germline nuclei or embryonic nuclei were defined as “Spot” objects. For germline quantifications, three nuclei within the progenitor zone (row 1 to row 8) were selected for further analysis per sample. The total amount of fluorescent intensity per nucleus was then taken using the intensity sum value (with background subtraction). For embryonic nuclei, cells bounded by AJM-1::GFP, specifically V3 to V5, were selected. The total amount of fluorescent intensity per nucleus was then taken using the intensity sum value (with background subtraction).

For each condition, a minimum of three biological replicates were performed. We analyzed the following number of embryonic nuclei in Fig. 3B: *his-72::Dendra2*: 2-cell (*n* = 9), 4-cell (*n* = 6), 20- to 50-cell (*n* = 12), 115- to 175-cell (*n* = 10), 230- to 300-cell (*n* = 22), and 350- to 400-cell (*n* = 21). *his-45::Dendra2*: 2-cell (*n* = 11), 4-cell (*n* = 29), 20- to 50-cell (*n* = 9), 115- to 175-cell (*n* = 18), 230- to 300-cell (*n* = 16), and 350- to 400-cell (*n* = 18). *his-6::Dendra2*: 20- to 50-cell (*n* = 6), 115- to 175-cell (*n* = 14), 230- to 300-cell (*n* = 17), and 350- to 400-cell (*n* = 18).

In Fig. 4 (B and D), for each condition, three biological replicates were performed. We analyzed the following number of samples per genotype: *his-45::Dendra2* (*n* = 3), *his-55::Dendra2* (*n* = 3), *his-59::Dendra2* (*n* = 3), *his-63::Dendra2* (*n* = 3), *his-2::Dendra2* (*n* = 3), *his-6::Dendra2* (*n* = 3), *his-45::Dendra2* (*n* = 3), *HIS4(H3::Dendra2)* (*n* = 3), *his-25::Dendra2* (*n* = 3), *his-32::Dendra2* (*n* = 3), *his-40::Dendra2* (*n* = 3), *his-72::GFP *(*n* = 3), and *his-71::GFP* (*n* = 3).

In Fig. 5 (C and D), for each condition, a minimum of three biological replicates were performed. The following number of embryonic nuclei were analyzed in both wild-type and *his-6(H113D)* embryos: wild-type: *his-55::*eGFP(H3), and *his-72*(H3.3)::mCherry at the 45- to 55-cell stage (*n* = 9, 9), 145- to 155-cell stage (*n* = 9, 9), 300- to 325-cell stage (*n* = 9, 9), and 395- to 415-cell stage (*n* = 9, 9); *his-6(H113D)*: *his-55::*eGFP(H3), *his-72*(H3.3)::mCherry at the 45- to 55-cell stage (*n* = 9, 9), 145- to 155-cell stage (*n* = 9, 9), 300- to 325-cell stage (*n* = 9, 9), and 395- to 415-cell stage (*n* = 9, 9).

In Fig. 6 (C and D), for each condition, a minimum of three biological replicates were performed. The following number of embryonic nuclei were analyzed in both wild-type and *his-6(H113D)* embryos: For wild-type, strain ST65 (*AJM-1::GFP)* was used to label seam cells at comma and 1.5-fold stages and stained for histone H4 in V5 (*n* = 21), V4 (*n* = 17), and V3 (*n* = 21) cells and H3K27me2/3 in V5 (*n* = 12), V4 (*n* = 8), and V3 (*n* = 11) cells. For *his-6(H113D)*, strain JHU79 {*ncIs13[AJM-1::GFP]*; *his-55 (kog11[his-55::TEV::eGFP::3xFlag])*; *his-72(kog5[his-72::mCherry])*; *his-6 (kog10)*} was used to label seam cells at comma and 1.5-fold stages and stained for histone H4 in V5 (*n* = 19), V4 (*n* = 18), and V3 (*n* = 19) cells and H3K27me2/3 in V5 (*n* = 13), V4 (*n* = 12), and V3 (*n* = 12) cells.

### Analysis software

Excel (Microsoft) and GraphPad Prism v9 were used for all data analysis and graphing. Images were captured with Zen Black (Zeiss) and Zen Blue (Zeiss). Fiji v1.53f was used for image processing and analysis. Imaris (Bitplane) v9.8.2 was used for image analysis. Photoshop (Adobe) and Illustrator (Adobe) were used for video editing and figure editing.

### Analysis of germline progenitor zone and pachytene nuclei

The strain GC1413 *rrf-1*(*pk1417)*; *naSi2* (*Pmex-5::H2B::mCherry::nos-2 3′UTR*); *teIs113* (*Ppie-1::GFP::H2B::zif-1 3′UTR*) was used to label all germline nuclei with mCherry (red), while progenitor zone nuclei are doubly marked with GFP and mCherry (yellow) in both wild-type and mutant backgrounds. Quantification of each region was measured by counting the rows of cells from the distal end (fig. S3A). For experiments where using this reporter was not possible because of overlap in the fluorescent channels, the germline regions were distinguished by row numbers based on the previously published analysis of the mitotic and meiotic regions (*92*, *93*). Specifically, the progenitor zone nuclei were selected for quantification from rows 1 to 5, the early pachytene nuclei were selected from rows 25 to 40, and the late pachytene nuclei were selected from rows 60 to 70 (Fig. 1F).

### Cell death assays

CED-1::GFP expressed in gonadal sheath cells was used to count engulfed germ line corpses as described previously (*50*). Strains containing CED-1::GFP in wild-type and mutant backgrounds were maintained at 20°C. L4 animals of each genotype were selected and, 24 hours later, scored for the number of engulfed apoptotic cells in the gonad.

### Brood size assays

Manually selected L4 animals were grown individually on petri dishes seeded with OP50 *E. coli* food until adulthood. They were then transferred on a new plate every 24 hours for a total of 5 days. The brood size of each worm was scored by counting the total number of larvae laid on the four plates. For each brood size experiment, at least 30 worms were scored for each strain.

### Cell fate challenge assay

Two-cell embryos were collected from wild-type and mutant backgrounds that carried the integrated reporter *otIs587 [gcy-5(fosmid)::SL2::NLS::GFP + ttx3p::mCherry]* and *otIs304 [hsp16-2p::che-1::3xHA::BLRP + rol-6(su1006)]*. Embryos were incubated for 480, 550, 670, and 760 min. Heat shock was administered at 32°C for 30 min, left at 20°C overnight, and scored about 24 hours later by counting the number of GFP-positive cells. In this assay, non-ASE cells that activate expression of *gcy-5* in response to *che-1* ectopic expression are considered plastic. Images were acquired with a Zeiss LSM 780 laser scanning confocal microscope.

We analyzed the following number of wild-type and *his-6(H113D)* embryos in Fig. 5F: wild-type: no heat shock (*n* = 22), *che-1* induced at 480 min (*n* = 24), *che-1* induced at 550 min (*n* = 51), *che-1* induced at 670 min (*n* = 40), and *che-1* induced at 760 min (*n* = 43); *His-6(H113D)*: no heat shock (*n* = 34), *che-1* induced at 480 min (*n* = 52), *che-1* induced at 550 min (*n* = 41), *che-1* induced at 670 min (*n* = 19), and *che-1* induced at 760 min (*n* = 28).

## Notes

View/request a protocol for this paper from *Bio-protocol*.

## References

[1] S. Strome, R. Lehmann, Germ versus soma decisions: Lessons from flies and worms. Science 316, 392–393 (2007). 17446385 10.1126/science.1140846

[2] S. H. Eun, Q. Gan, X. Chen, Epigenetic regulation of germ cell differentiation. Curr. Opin. Cell Biol. 22, 737–743 (2010). 20951019 10.1016/j.ceb.2010.09.004 PMC2993805

[3] A. Gaspar-Maia, A. Alajem, E. Meshorer, M. Ramalho-Santos, Open chromatin in pluripotency and reprogramming. Nat. Rev. Mol. Cell Biol. 12, 36–47 (2011). 21179060 10.1038/nrm3036 PMC3891572

[4] P. Meister, S. E. Mango, S. M. Gasser, Locking the genome: Nuclear organization and cell fate. Curr. Opin. Genet. Dev. 21, 167–174 (2011). 21345665 10.1016/j.gde.2011.01.023 PMC4041333

[5] P. Meister, S. Schott, C. Bedet, Y. Xiao, S. Rohner, S. Bodennec, B. Hudry, L. Molin, F. Solari, S. M. Gasser, F. Palladino, *Caenorhabditis elegans* heterochromatin protein 1 (HPL-2) links developmental plasticity, longevity and lipid metabolism. Genome Biol. 12, R123 (2011). 22185090 10.1186/gb-2011-12-12-r123 PMC3334618

[6] T. Yuzyuk, T. H. Fakhouri, J. Kiefer, S. E. Mango, The polycomb complex protein mes-2/E(z) promotes the transition from developmental plasticity to differentiation in *C. elegans* embryos. Dev. Cell 16, 699–710 (2009). 19460346 10.1016/j.devcel.2009.03.008 PMC2693235

[7] B. Mutlu, H.-M. Chen, S. Gutnik, D. H. Hall, S. Keppler-Ross, S. E. Mango, Distinct functions and temporal regulation of methylated histone H3 during early embryogenesis. Development 146, dev174516 (2019). 31540912 10.1242/dev.174516 PMC6803369

[8] D. Filipescu, S. Muller, G. Almouzni, Histone H3 variants and their chaperones during development and disease: Contributing to epigenetic control. Annu. Rev. Cell Dev. Biol. 30, 615–646 (2014). 25288118 10.1146/annurev-cellbio-100913-013311

[9] D. Filipescu, E. Szenker, G. Almouzni, Developmental roles of histone H3 variants and their chaperones. Trends Genet. 29, 630–640 (2013). 23830582 10.1016/j.tig.2013.06.002

[10] H. Tagami, D. Ray-Gallet, G. Almouzni, Y. Nakatani, Histone H3.1 and H3.3 complexes mediate nucleosome assembly pathways dependent or independent of DNA synthesis. Cell 116, 51–61 (2004). 14718166 10.1016/s0092-8674(03)01064-x

[11] E. Szenker, D. Ray-Gallet, G. Almouzni, The double face of the histone variant H3.3. Cell Res. 21, 421–434 (2011). 21263457 10.1038/cr.2011.14 PMC3193428

[12] D. M. Truong, J. D. Boeke, Resetting the yeast epigenome with human nucleosomes. Cell 171, 1508–1519.e13 (2017). 29198523 10.1016/j.cell.2017.10.043 PMC5732057

[13] W. F. Marzluff, E. J. Wagner, R. J. Duronio, Metabolism and regulation of canonical histone mRNAs: Life without a poly(A) tail. Nat. Rev. Genet. 9, 843–854 (2008). 18927579 10.1038/nrg2438 PMC2715827

[14] W. F. Marzluff, P. Gongidi, K. R. Woods, J. Jin, L. J. Maltais, The human and mouse replication-dependent histone genes. Genomics 80, 487–498 (2002). 12408966

[15] H. S. Malik, S. Henikoff, Phylogenomics of the nucleosome. Nat. Struct. Biol. 10, 882–891 (2003). 14583738 10.1038/nsb996

[16] H. Tachiwana, A. Osakabe, T. Shiga, Y. Miya, H. Kimura, W. Kagawa, H. Kurumizaka, Structures of human nucleosomes containing major histone H3 variants. Acta Crystallogr. D Biol. Crystallogr. 67, 578–583 (2011). 21636898 10.1107/S0907444911014818

[17] S. Mendiratta, A. Gatto, G. Almouzni, Histone supply: Multitiered regulation ensures chromatin dynamics throughout the cell cycle. J. Cell Biol. 218, 39–54 (2019). 30257851 10.1083/jcb.201807179 PMC6314538

[18] C. Clement, G. A. Orsi, A. Gatto, E. Boyarchuk, A. Forest, B. Hajj, J. Miné-Hattab, M. Garnier, Z. A. Gurard-Levin, J.-P. Quivy, G. Almouzni, High-resolution visualization of H3 variants during replication reveals their controlled recycling. Nat. Commun. 9, 3181 (2018). 30093638 10.1038/s41467-018-05697-1 PMC6085313

[19] A. D. Goldberg, L. A. Banaszynski, K.-M. Noh, P. W. Lewis, S. J. Elsaesser, S. Stadler, S. Dewell, M. Law, X. Guo, X. Li, D. Wen, A. Chapgier, R. C. De Kelver, J. C. Miller, Y.-L. Lee, E. A. Boydston, M. C. Holmes, P. D. Gregory, J. M. Greally, S. Rafii, C. Yang, P. J. Scambler, D. Garrick, R. J. Gibbons, D. R. Higgs, I. M. Cristea, F. D. Urnov, D. Zheng, C. D. Allis, Distinct factors control histone variant H3.3 localization at specific genomic regions. Cell 140, 678–691 (2010). 20211137 10.1016/j.cell.2010.01.003 PMC2885838

[20] P. W. Lewis, S. J. Elsaesser, K. M. Noh, S. C. Stadler, C. D. Allis, Daxx is an H3.3-specific histone chaperone and cooperates with ATRX in replication-independent chromatin assembly at telomeres. Proc. Natl. Acad. Sci. U.S.A. 107, 14075–14080 (2010). 20651253 10.1073/pnas.1008850107 PMC2922592

[21] D. Ray-Gallet, G. Almouzni, The histone H3 family and its deposition pathways. Adv. Exp. Med. Biol. 1283, 17–42 (2021). 33155135 10.1007/978-981-15-8104-5_2

[22] E. McKittrick, P. R. Gafken, K. Ahmad, S. Henikoff, Histone H3.3 is enriched in covalent modifications associated with active chromatin. Proc. Natl. Acad. Sci. U.S.A. 101, 1525–1530 (2004). 14732680 10.1073/pnas.0308092100 PMC341768

[23] A. Loyola, T. Bonaldi, D. Roche, A. Imhof, G. Almouzni, PTMs on H3 variants before chromatin assembly potentiate their final epigenetic state. Mol. Cell 24, 309–316 (2006). 17052464 10.1016/j.molcel.2006.08.019

[24] S. B. Hake, C. D. Allis, Histone H3 variants and their potential role in indexing mammalian genomes: The "H3 barcode hypothesis". Proc. Natl. Acad. Sci. U.S.A. 103, 6428–6435 (2006). 16571659 10.1073/pnas.0600803103 PMC1564199

[25] S. B. Hake, B. A. Garcia, E. M. Duncan, M. Kauer, G. Dellaire, J. Shabanowitz, D. P. Bazett-Jones, C. D. Allis, D. F. Hunt, Expression patterns and post-translational modifications associated with mammalian histone H3 variants. J. Biol. Chem. 281, 559–568 (2006). 16267050 10.1074/jbc.M509266200

[26] J. Kreher, T. Takasaki, C. Cockrum, S. Sidoli, B. A. Garcia, O. N. Jensen, S. Strome, Distinct roles of two histone methyltransferases in transmitting H3K36me3-based epigenetic memory across generations in *Caenorhabditis elegans*. Genetics 210, 969–982 (2018). 30217796 10.1534/genetics.118.301353 PMC6218224

[27] C. Couldrey, M. B. Carlton, P. M. Nolan, W. H. Colledge, M. J. Evans, A retroviral gene trap insertion into the histone 3.3A gene causes partial neonatal lethality, stunted growth, neuromuscular deficits and male sub-fertility in transgenic mice. Hum. Mol. Genet. 8, 2489–2495 (1999). 10556297 10.1093/hmg/8.13.2489

[28] M. C. Tang, S. A. Jacobs, D. M. Mattiske, Y. M. Soh, A. N. Graham, A. Tran, S. L. Lim, D. F. Hudson, P. Kalitsis, M. K. O’Bryan, L. H. Wong, J. R. Mann, Contribution of the two genes encoding histone variant h3.3 to viability and fertility in mice. PLOS Genet. 11, e1004964 (2015). 25675407 10.1371/journal.pgen.1004964 PMC4335506

[29] K. M. Bush, B. T. Yuen, B. L. Barrilleaux, J. W. Riggs, H. O'Geen, R. F. Cotterman, P. S. Knoepfler, Endogenous mammalian histone H3.3 exhibits chromatin-related functions during development. Epigenetics Chromatin 6, 7 (2013). 23570311 10.1186/1756-8935-6-7 PMC3635903

[30] C. W. Jang, Y. Shibata, J. Starmer, D. Yee, T. Magnuson, Histone H3.3 maintains genome integrity during mammalian development. Genes Dev 29, 1377–1392 (2015). 26159997 10.1101/gad.264150.115 PMC4511213

[31] M. Hodl, K. Basler, Transcription in the absence of histone H3.3. Curr. Biol. 19, 1221–1226 (2009). 19523831 10.1016/j.cub.2009.05.048

[32] A. Sakai, B. E. Schwartz, S. Goldstein, K. Ahmad, Transcriptional and developmental functions of the H3.3 histone variant in *Drosophila*. Curr. Biol. 19, 1816–1820 (2009). 19781938 10.1016/j.cub.2009.09.021 PMC2783816

[33] B. Loppin, E. Bonnefoy, C. Anselme, A. Laurençon, T. L. Karr, P. Couble, The histone H3.3 chaperone HIRA is essential for chromatin assembly in the male pronucleus. Nature 437, 1386–1390 (2005). 16251970 10.1038/nature04059

[34] E. Szenker, N. Lacoste, G. Almouzni, A developmental requirement for HIRA-dependent H3.3 deposition revealed at gastrulation in *Xenopus*. Cell Rep. 1, 730–740 (2012). 22813747 10.1016/j.celrep.2012.05.006

[35] K. Delaney, J. Mailler, J. M. Wenda, C. Gabus, F. A. Steiner, Differential expression of histone H3.3 genes and their role in modulating temperature stress response in *Caenorhabditis elegans*. Genetics 209, 551–565 (2018). 29636369 10.1534/genetics.118.300909 PMC5972426

[36] A. Paix, A. Folkmann, G. Seydoux, Precision genome editing using CRISPR-Cas9 and linear repair templates in *C. elegans*. Methods 121–122, 86–93 (2017). 10.1016/j.ymeth.2017.03.023 PMC6788293 28392263

[37] H. Bukhari, T. Muller, Endogenous fluorescence tagging by CRISPR. Trends Cell Biol. 29, 912–928 (2019). 31522960 10.1016/j.tcb.2019.08.004

[38] A. Paix, Y. Wang, H. E. Smith, C. Y. S. Lee, D. Calidas, T. Lu, J. Smith, H. Schmidt, M. W. Krause, G. Seydoux, Scalable and versatile genome editing using linear DNAs with microhomology to Cas9 sites in *Caenorhabditis elegans*. Genetics 198, 1347–1356 (2014). 25249454 10.1534/genetics.114.170423 PMC4256755

[39] M. E. Boeck, C. Huynh, L. Gevirtzman, O. A. Thompson, G. Wang, D. M. Kasper, V. Reinke, L. D. W. Hillier, R. H. Waterston, The time-resolved transcriptome of *C. elegans*. Genome Res. 26, 1441–1450 (2016). 27531719 10.1101/gr.202663.115 PMC5052054

[40] L. B. Bender, R. Cao, Y. Zhang, S. Strome, The MES-2/MES-3/MES-6 complex and regulation of histone H3 methylation in *C. elegans*. Curr. Biol. 14, 1639–1643 (2004). 15380065 10.1016/j.cub.2004.08.062

[41] L. B. Bender, J. Suh, C. R. Carroll, Y. Fong, I. M. Fingerman, S. D. Briggs, R. Cao, Y. Zhang, V. Reinke, S. Strome, MES-4: An autosome-associated histone methyltransferase that participates in silencing the X chromosomes in the *C. elegans* germ line. Development 133, 3907–3917 (2006). 16968818 10.1242/dev.02584 PMC2435371

[42] C. E. Schaner, W. G. Kelly, Germline chromatin. WormBook , 1–14 (2006). 10.1895/wormbook.1.73.1 PMC4781259 18050477

[43] S. L. Ooi, S. Henikoff, Germline histone dynamics and epigenetics. Curr. Opin. Cell Biol. 19, 257–265 (2007). 17467256 10.1016/j.ceb.2007.04.015

[44] J. K. Arico, D. J. Katz, J. van der Vlag, W. G. Kelly, Epigenetic patterns maintained in early *Caenorhabditis elegans* embryos can be established by gene activity in the parental germ cells. PLOS Genet. 7, e1001391 (2011). 21695223 10.1371/journal.pgen.1001391 PMC3111476

[45] M. Sivaguru, M. A. Urban, G. Fried, C. J. Wesseln, L. Mander, S. W. Punyasena, Comparative performance of airyscan and structured illumination superresolution microscopy in the study of the surface texture and 3D shape of pollen. Microsc. Res. Tech. 81, 101–114 (2018). 27476493 10.1002/jemt.22732

[46] J. E. Sulston, E. Schierenberg, J. G. White, J. N. Thomson, The embryonic cell lineage of the nematode *Caenorhabditis elegans*. Dev. Biol. 100, 64–119 (1983). 6684600 10.1016/0012-1606(83)90201-4

[47] I. E. Schauer, W. B. Wood, Early *C. elegans* embryos are transcriptionally active. Development 110, 1303–1317 (1990). 2100265 10.1242/dev.110.4.1303

[48] G. Seydoux, A. Fire, Soma-germline asymmetry in the distributions of embryonic RNAs in *Caenorhabditis elegans*. Development 120, 2823–2834 (1994). 7607073 10.1242/dev.120.10.2823

[49] D. Roy, D. J. Kahler, C. Yun, E. J. A. Hubbard, Functional interactions between rsks-1/S6K, glp-1/Notch, and regulators of *Caenorhabditis elegans* fertility and germline stem cell maintenance. G3 8, 3293–3309 (2018). 30126834 10.1534/g3.118.200511 PMC6169383

[50] Z. Zhou, E. Hartwieg, H. R. Horvitz, CED-1 is a transmembrane receptor that mediates cell corpse engulfment in *C. elegans*. Cell 104, 43–56 (2001). 11163239 10.1016/s0092-8674(01)00190-8

[51] C. Garvin, R. Holdeman, S. Strome, The phenotype of mes-2, mes-3, mes-4 and mes-6, maternal-effect genes required for survival of the germline in *Caenorhabditis elegans*, is sensitive to chromosome dosage. Genetics 148, 167–185 (1998). 9475730 10.1093/genetics/148.1.167 PMC1459798

[52] A. N. McMurchy, P. Stempor, T. Gaarenstroom, B. Wysolmerski, Y. Dong, D. Aussianikava, A. Appert, N. Huang, P. Kolasinska-Zwierz, A. Sapetschnig, E. A. Miska, J. Ahringer, A team of heterochromatin factors collaborates with small RNA pathways to combat repetitive elements and germline stress. eLife 6, (2017). 10.7554/eLife.21666 PMC5395297 28294943

[53] A. N. Sawh, M. E. R. Shafer, J.-H. Su, X. Zhuang, S. Wang, S. E. Mango, Lamina-dependent stretching and unconventional chromosome compartments in Early *C. elegans* embryos. Mol. Cell 78, 96–111.e6 (2020). 32105612 10.1016/j.molcel.2020.02.006 PMC7263362

[54] J. Rothman, S. Jarriault, Developmental plasticity and cellular reprogramming in *Caenorhabditis elegans*. Genetics 213, 723–757 (2019). 31685551 10.1534/genetics.119.302333 PMC6827377

[55] B. Mutlu, H.-M. Chen, J. J. Moresco, B. D. Orelo, B. Yang, J. M. Gaspar, S. Keppler-Ross, J. R. Yates III, D. H. Hall, E. M. Maine, S. E. Mango, Regulated nuclear accumulation of a histone methyltransferase times the onset of heterochromatin formation in *C. elegans* embryos. Sci. Adv. 4, eaat6224 (2018). 30140741 10.1126/sciadv.aat6224 PMC6105299

[56] T. Patel, O. Hobert, Coordinated control of terminal differentiation and restriction of cellular plasticity. eLife 6, (2017). 10.7554/eLife.24100 PMC5397285 28422646

[57] J. Hardy, D. Dai, A. Ait Saada, A. Teixeira-Silva, L. Dupoiron, F. Mojallali, K. Fréon, F. Ochsenbein, B. Hartmann, S. Lambert, Histone deposition promotes recombination-dependent replication at arrested forks. PLOS Genet. 15, e1008441 (2019). 31584934 10.1371/journal.pgen.1008441 PMC6795475

[58] S. Nakano, B. Stillman, H. R. Horvitz, Replication-coupled chromatin assembly generates a neuronal bilateral asymmetry in *C. elegans*. Cell 147, 1525–1536 (2011). 22177093 10.1016/j.cell.2011.11.053 PMC3290763

[59] E. A. Spickard, P. M. Joshi, J. H. Rothman, The multipotency-to-commitment transition in *Caenorhabditis elegans*-implications for reprogramming from cells to organs. FEBS Lett. 592, 838–851 (2018). 29334121 10.1002/1873-3468.12977 PMC6385892

[60] J. S. Gilleard, J. D. McGhee, Activation of hypodermal differentiation in the *Caenorhabditis elegans* embryo by GATA transcription factors ELT-1 and ELT-3. Mol. Cell. Biol. 21, 2533–2544 (2001). 11259601 10.1128/MCB.21.7.2533-2544.2001 PMC86885

[61] J. Zhu, T. Fukushige, J. D. McGhee, J. H. Rothman, Reprogramming of early embryonic blastomeres into endodermal progenitors by a *Caenorhabditis elegans* GATA factor. Genes Dev. 12, 3809–3814 (1998). 9869634 10.1101/gad.12.24.3809 PMC317268

[62] T. Fukushige, M. Krause, The myogenic potency of HLH-1 reveals wide-spread developmental plasticity in early *C. elegans* embryos. Development 132, 1795–1805 (2005). 15772130 10.1242/dev.01774

[63] N. J. Djabrayan, N. R. Dudley, E. M. Sommermann, J. H. Rothman, Essential role for Notch signaling in restricting developmental plasticity. Genes Dev. 26, 2386–2391 (2012). 23124064 10.1101/gad.199588.112 PMC3489997

[64] J. R. Priess, J. N. Thomson, Cellular interactions in early *C. elegans* embryos. Cell 48, 241–250 (1987). 3802194 10.1016/0092-8674(87)90427-2

[65] M. A. Horner, S. Quintin, M. E. Domeier, J. Kimble, M. Labouesse, S. E. Mango, pha-4, an HNF-3 homolog, specifies pharyngeal organ identity in *Caenorhabditis elegans*. Genes Dev. 12, 1947–1952 (1998). 9649499 10.1101/gad.12.13.1947 PMC316969

[66] S. Chang, R. J. Johnston Jr., O. Hobert, A transcriptional regulatory cascade that controls left/right asymmetry in chemosensory neurons of *C. elegans*. Genes Dev. 17, 2123–2137 (2003). 12952888 10.1101/gad.1117903 PMC196454

[67] O. Uchida, H. Nakano, M. Koga, Y. Ohshima, The *C. elegans* che-1 gene encodes a zinc finger transcription factor required for specification of the ASE chemosensory neurons. Development 130, 1215–1224 (2003). 12588839 10.1242/dev.00341

[68] B. Tursun, T. Patel, P. Kratsios, O. Hobert, Direct conversion of *C. elegans* germ cells into specific neuron types. Science 331, 304–308 (2011). 21148348 10.1126/science.1199082 PMC3250927

[69] J. F. Etchberger, A. Lorch, M. C. Sleumer, R. Zapf, S. J. Jones, M. A. Marra, R. A. Holt, D. G. Moerman, O. Hobert, The molecular signature and cis-regulatory architecture of a *C. elegans* gustatory neuron. Genes Dev. 21, 1653–1674 (2007). 17606643 10.1101/gad.1560107 PMC1899474

[70] D. Ray-Gallet, A. Woolfe, I. Vassias, C. Pellentz, N. Lacoste, A. Puri, D. C. Schultz, N. A. Pchelintsev, P. D. Adams, L. E. T. Jansen, G. Almouzni, Dynamics of histone H3 deposition in vivo reveal a nucleosome gap-filling mechanism for H3.3 to maintain chromatin integrity. Mol. Cell 44, 928–941 (2011). 22195966 10.1016/j.molcel.2011.12.006

[71] L. Cheng, X. Zhang, Y. Wang, H. Gan, X. Xu, X. Lv, X. Hua, J. Que, T. Ordog, Z. Zhang, Chromatin assembly factor 1 (CAF-1) facilitates the establishment of facultative heterochromatin during pluripotency exit. Nucleic Acids Res. 47, 11114–11131 (2019). 31586391 10.1093/nar/gkz858 PMC6868363

[72] D. Jiang, F. Berger, DNA replication-coupled histone modification maintains polycomb gene silencing in plants. Science 357, 1146–1149 (2017). 28818970 10.1126/science.aan4965

[73] Z. F. Wang, T. Krasikov, M. R. Frey, J. Wang, A. G. Matera, W. F. Marzluff, Characterization of the mouse histone gene cluster on chromosome 13: 45 histone genes in three patches spread over 1Mb. Genome Res. 6, 688–701 (1996). 8858344 10.1101/gr.6.8.688

[74] R. Maxson, R. Cohn, L. Kedes, T. Mohun, Expression and organization of histone genes. Annu. Rev. Genet. 17, 239–277 (1983). 6421226 10.1146/annurev.ge.17.120183.001323

[75] G. Romano, Histone variants during sea urchin development. Cell Biol. Int. Rep. 16, 197–206 (1992). 1581966 10.1016/s0309-1651(06)80121-9

[76] G. Childs, R. Maxson, L. H. Kedes, Histone gene expression during sea urchin embryogenesis: Isolation and characterization of early and late messenger RNAs of *Strongylocentrotus purpuratus* by gene-specific hybridization and template activity. Dev. Biol. 73, 153–173 (1979). 527767 10.1016/0012-1606(79)90144-1

[77] A. R. Strom, A. V. Emelyanov, M. Mir, D. V. Fyodorov, X. Darzacq, G. H. Karpen, Phase separation drives heterochromatin domain formation. Nature 547, 241–245 (2017). 28636597 10.1038/nature22989 PMC6022742

[78] K. Laue, S. Rajshekar, A. J. Courtney, Z. A. Lewis, M. G. Goll, The maternal to zygotic transition regulates genome-wide heterochromatin establishment in the zebrafish embryo. Nat. Commun. 10, 1551 (2019). 30948728 10.1038/s41467-019-09582-3 PMC6449393

[79] K. Ahmed, H. Dehghani, P. Rugg-Gunn, E. Fussner, J. Rossant, D. P. Bazett-Jones, Global chromatin architecture reflects pluripotency and lineage commitment in the early mouse embryo. PLOS ONE 5, e10531 (2010). 20479880 10.1371/journal.pone.0010531 PMC2866533

[80] S. Matoba, Y. Liu, F. Lu, K. A. Iwabuchi, L. Shen, A. Inoue, Y. Zhang, Embryonic development following somatic cell nuclear transfer impeded by persisting histone methylation. Cell 159, 884–895 (2014). 25417163 10.1016/j.cell.2014.09.055 PMC4243038

[81] B. Wen, H. Wu, Y. Shinkai, R. A. Irizarry, A. P. Feinberg, Large histone H3 lysine 9 dimethylated chromatin blocks distinguish differentiated from embryonic stem cells. Nat. Genet. 41, 246–250 (2009). 19151716 10.1038/ng.297 PMC2632725

[82] Y. G. Chung, S. Matoba, Y. Liu, J. H. Eum, F. Lu, W. Jiang, J. E. Lee, V. Sepilian, K. Y. Cha, D. R. Lee, Y. Zhang, Histone demethylase expression enhances human somatic cell nuclear transfer efficiency and promotes derivation of pluripotent stem cells. Cell Stem Cell 17, 758–766 (2015). 26526725 10.1016/j.stem.2015.10.001

[83] A. R. Cutter DiPiazza, N. Taneja, J. Dhakshnamoorthy, D. Wheeler, S. Holla, S. I. S. Grewal, Spreading and epigenetic inheritance of heterochromatin require a critical density of histone H3 lysine 9 tri-methylation. Proc. Natl. Acad. Sci. U.S.A. 118, (2021). 10.1073/pnas.2100699118 PMC8179192 34035174

[84] T. Ishiuchi, S. Abe, K. Inoue, W. K. A. Yeung, Y. Miki, A. Ogura, H. Sasaki, Reprogramming of the histone H3.3 landscape in the early mouse embryo. Nat. Struct. Mol. Biol. 28, 38–49 (2021). 33169018 10.1038/s41594-020-00521-1

[85] W. Xia, J. Xu, G. Yu, G. Yao, K. Xu, X. Ma, N. Zhang, B. Liu, T. Li, Z. Lin, X. Chen, L. Li, Q. Wang, D. Shi, S. Shi, Y. Zhang, W. Song, H. Jin, L. Hu, Z. Bu, Y. Wang, J. Na, W. Xie, Y. P. Sun, Resetting histone modifications during human parental-to-zygotic transition. Science 365, 353–360 (2019). 31273069 10.1126/science.aaw5118

[86] L. J. Gaydos, W. Wang, S. Strome, Gene repression. H3K27me and PRC2 transmit a memory of repression across generations and during development. Science 345, 1515–1518 (2014). 25237104 10.1126/science.1255023 PMC4238426

[87] A. Inoue, L. Jiang, F. Lu, Y. Zhang, Genomic imprinting of Xist by maternal H3K27me3. Genes Dev. 31, 1927–1932 (2017). 29089420 10.1101/gad.304113.117 PMC5710138

[88] S. Brenner, The genetics of *Caenorhabditis elegans*. Genetics 77, 71–94 (1974). 4366476 10.1093/genetics/77.1.71 PMC1213120

[89] S. L. Ooi, J. R. Priess, S. Henikoff, Histone H3.3 variant dynamics in the germline of *Caenorhabditis elegans*. PLOS Genet. 2, e97 (2006). 16846252 10.1371/journal.pgen.0020097 PMC1484599

[90] B. E. Schwartz, K. Ahmad, Transcriptional activation triggers deposition and removal of the histone variant H3.3. Genes Dev. 19, 804–814 (2005). 15774717 10.1101/gad.1259805 PMC1074318

[91] J. Schindelin, I. Arganda-Carreras, E. Frise, V. Kaynig, M. Longair, T. Pietzsch, S. Preibisch, C. Rueden, S. Saalfeld, B. Schmid, J. Y. Tinevez, D. J. White, V. Hartenstein, K. Eliceiri, P. Tomancak, A. Cardona, Fiji: An open-source platform for biological-image analysis. Nat. Methods 9, 676–682 (2012). 22743772 10.1038/nmeth.2019 PMC3855844

[92] C. Lee, E. B. Sorensen, T. R. Lynch, J. Kimble, *C. elegans* GLP-1/notch activates transcription in a probability gradient across the germline stem cell pool. eLife 5, e18370 (2016). 27705743 10.7554/eLife.18370 PMC5094854

[93] S. L. Crittenden, H. S. Seidel, J. Kimble, Analysis of the *C. elegans* germline stem cell pool. Methods Mol. Biol. 1463, 1–33 (2017). 27734344 10.1007/978-1-4939-4017-2_1

