# Supplementary material for: Developmentally programmed histone H3 expression regulates cellular plasticity at the parental-to-early embryo transition
Source: Sci Adv. 2023 Apr 7;9(14):eadh0411. doi: 10.1126/sciadv.adh0411 (PMC10081851; doi:10.1126/sciadv.adh0411)
Supplement: Supplementary file 1 — Figs. S1 to S3 Tables S1 to S3 Legends for movies S1 to S3 [file sciadv.adh0411_sm.pdf]

Science Advances
AAAS

# Supplementary Materials for

## Developmentally programmed histone H3 expression regulates cellular plasticity at the parental-to-early embryo transition

Ryan J. Gleason *et al.*

Corresponding author: Ryan J. Gleason, rygleason@jhu.edu; Xin Chen, xchen32@jhu.edu

*Sci. Adv.* **9**, eadh0411 (2023)
DOI: 10.1126/sciadv.adh0411

**The PDF file includes:**

Figs. S1 to S3
Tables S1 to S3
Legends for movies S1 to S3

**Other Supplementary Material for this manuscript includes the following:**

Movies S1 to S3

## Supplemental Materials

## Supplementary Figures and Figure Legends:

**Figure S1. Expression patterns of all histone *H3* gene clusters in *C. elegans* adult hermaphrodites.**

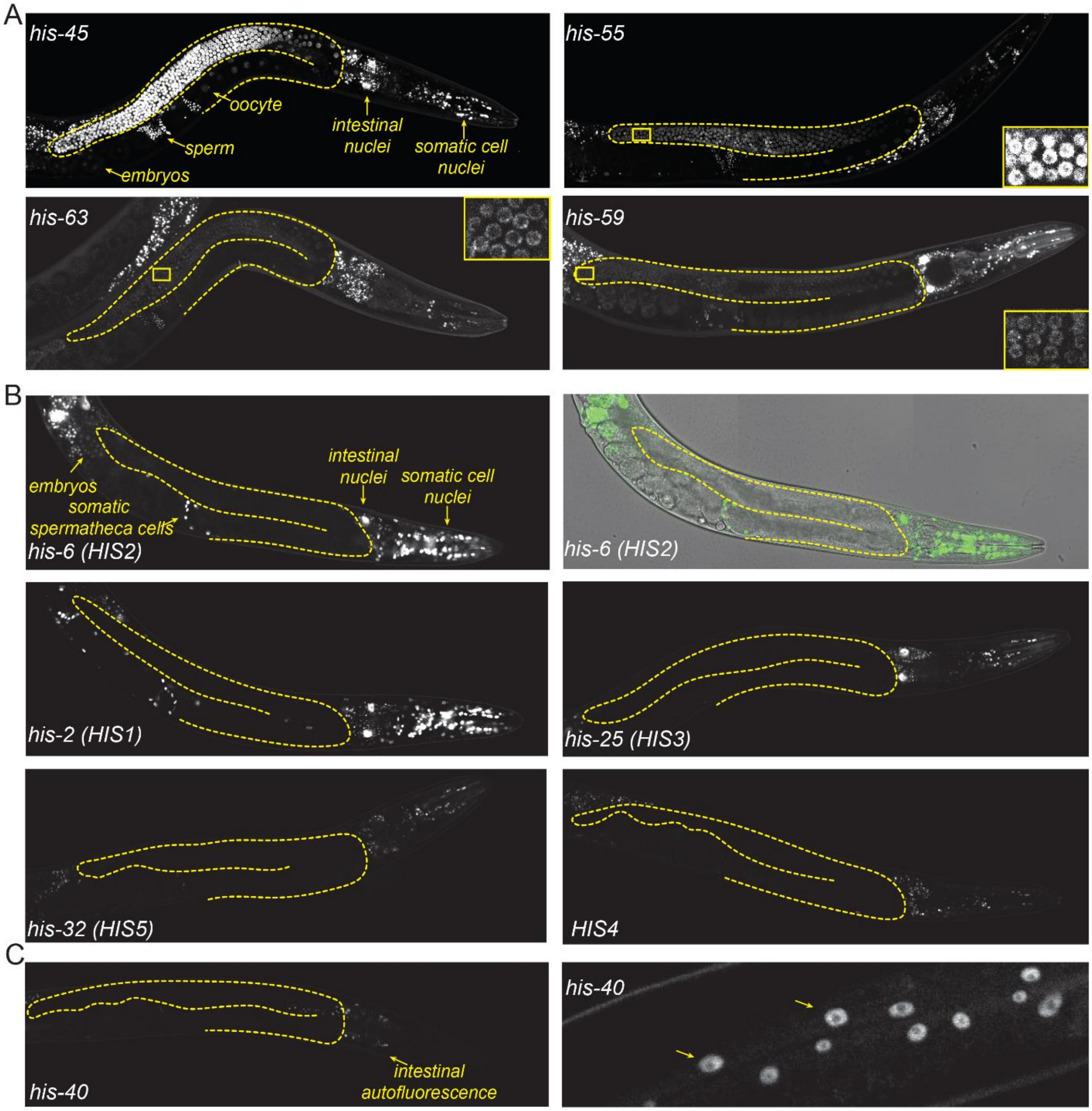

**Figure S1 legend: Expression patterns of all histone *H3* gene clusters in *C. elegans* adult hermaphrodites.** (**A**) Representative fluorescence micrographs of ubiquitously expressed Class I histone *H3* isotypes including *his-45, his-55, his-63,* and *his-59*. The dashed lines outline the gonads, and distinct cell types are marked as an example. Insets demonstrate that *his-55, his-63,* and *his-59* are detectable in the germline by increasing the brightness. (**B**) Representative fluorescence micrographs for one member of each of the five histone gene clusters including HIS1 (*his-2*), HIS2 (*his-6*), HIS3 (*his-25*), HIS4 (*his-17*, *his-27*, and/or *his-49,* see methods for details), and HIS5 (*his-32*). Histone *H3* isotypes encoded in HIS1-5 are detectable in all somatic lineages, but undetectable in the germline. (**C**) *his-40* encodes a histone H3 that is detectable in epithelial nuclei including the hypodermis (epidermis) marked by yellow arrows.

**Figure S2. HIS-71(H3.3)::GFP is detectable at late pachytene as nuclei transition from pachytene to diakinesis and initiate oocyte formation.**

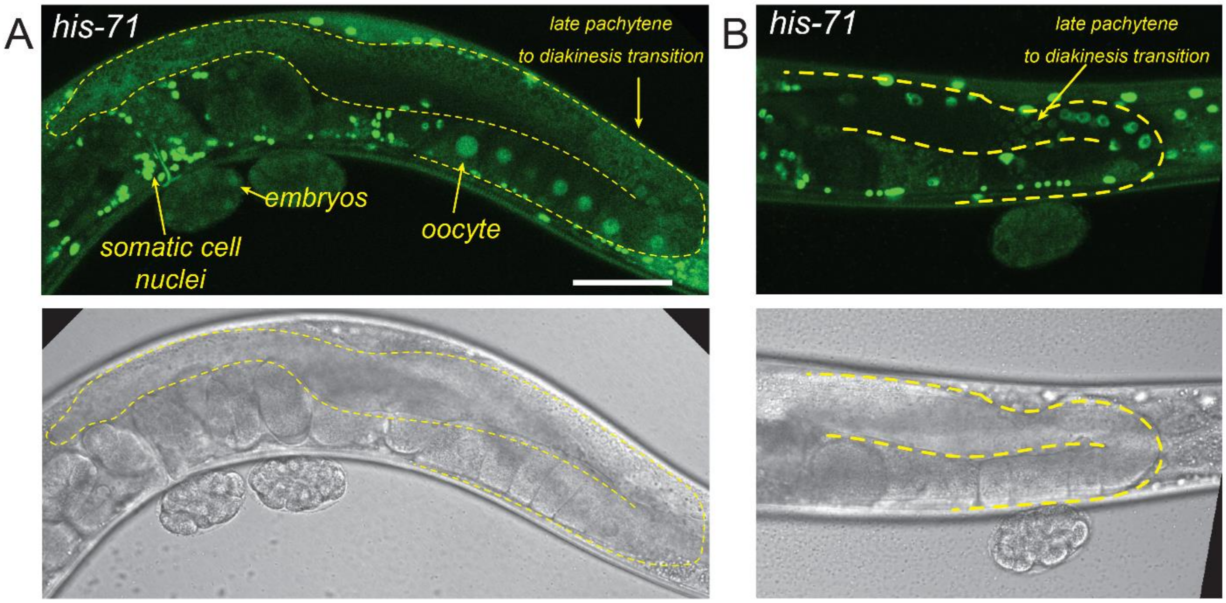

**Figure S2 legend:** (A) Expression pattern of an endogenously tagged GFP fusion strain for *his-71 (H3.3),* GFP (top), and DIC (bottom). HIS-71::GFP is undetectable in the mitotic and early meiotic pachytene regions of the germline. Expression of HIS-71::GFP is observed as nuclei transition into the loop region of the germline, where they transition from pachytene to diakinesis and initiate oocyte formation. The dashed lines outline the gonads, and distinct cell types are marked as an example, including oocytes, early stage embryos, and somatic cells. Somatic cells are notably higher in his-71 expression. (B) A second sample of HIS-71::GFP, which is positioned in an orientation optimal for detecting nuclei expression in the loop region of the germline, GFP (top), and DIC (bottom).

**Figure S3. Knockouts of the germline-expressed histone *H3* genes lead to decreased fecundity and germ cell nuclei, as well as increased germline apoptosis.**

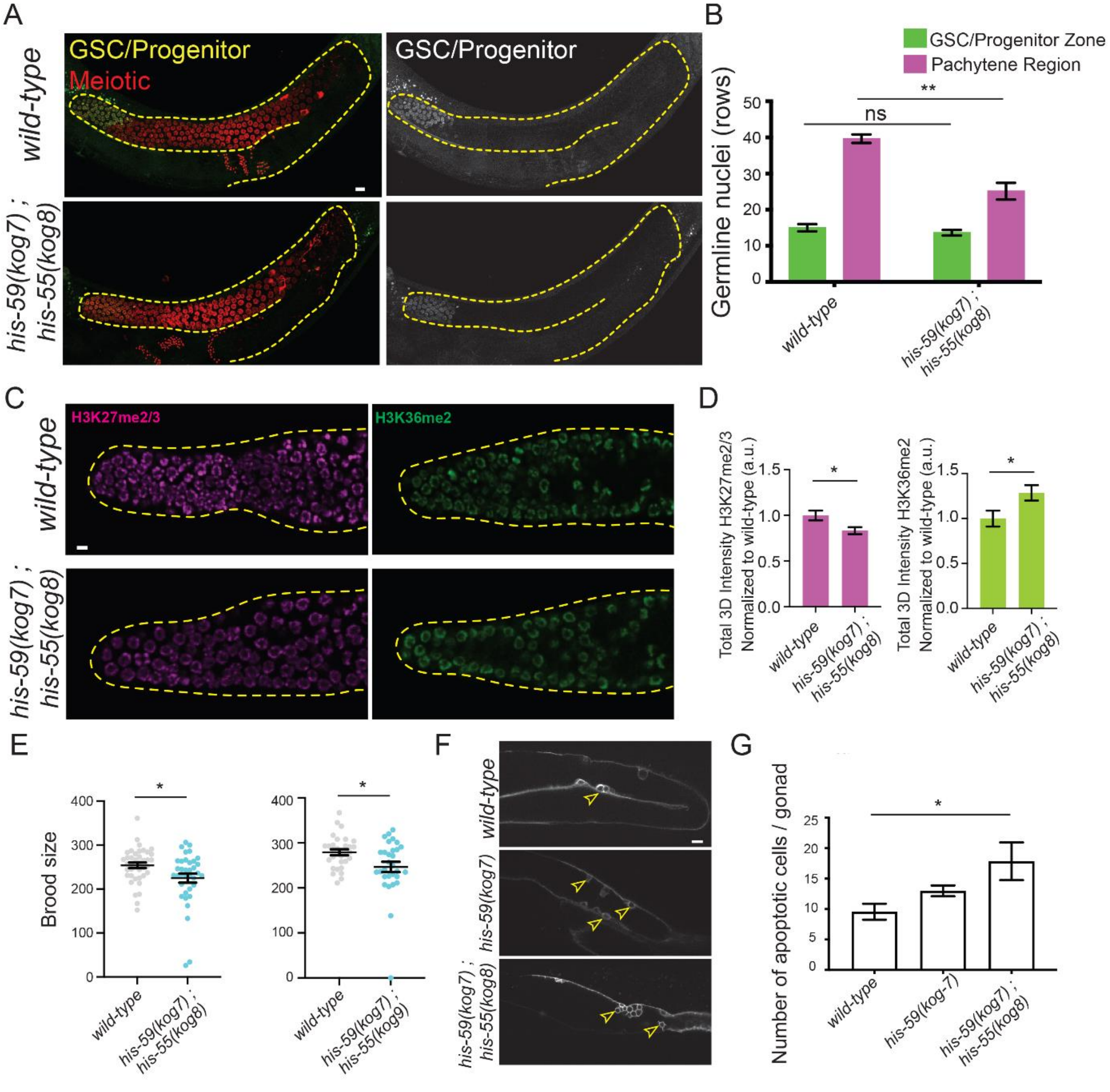

**Figure S3 legend:** (**A**) Representative images of wild-type and *his-59(kog7); his-55(kog8)* double mutant of histone *H3* genes. The strain GC1413 *rrf-1*(*pk1417*; *naSi2* (*Pmex-5::H2B::mCherry::nos-2 3'UTR*); *teIs113* (*Ppie-1::GFP::H2B::zif-1 3'UTR*)) was used to label all germline nuclei with mCherry (red), while progenitor nuclei are doubly marked with GFP and mCherry (yellow). The dashed lines outline the gonads. (**B**) Quantification measured by rows of cells from the distal end. All quantifications = average ± SE. *P*-value: unpaired t test, showing no significant difference ($P$ = 0.3192) in the Progenitor region (GFP- and mCherry-double positive germ cells) of *his-59(kog7);his-55(kog8)* double histone *H3* mutant (n=4) and wild-type (n=3), but a significant difference (**$P$ = 0.0054) in the pachytene region (GFP-negative and mCherry-positive germ cells), *his-59(kog7);his-55(kog8)* double histone *H3* mutant (n=8) and wild-type (n=8). (**C**) Immunofluorescent micrographs of wild-type and double histone H3 mutant, *his-59(kog7);his-55(kog8),* stained for H3K27me2/3 (magenta) or H3K36me2 (green). (**D**) Quantification of total 3D intensity of either H3K27me2/3 (*$P$ = 0.0133) or H3K36me2 (*$P$ = 0.0240) of data sets from (C). (**E**) Brood sizes for two double histone H3 mutant strains including *his-59(kog7); his-55(kog8)* (n=36) and wild-type (n=39) (*$P$ = 0.0171), and *his-59(kog7); his-55(kog9*) (n=30) and wild-type (n=30) (*$P$ = 0.0181). Each data point represents the number of living larvae from individual worms with the corresponding genotype. (**F**) Representative images of nuclei undergoing programmed cell death marked by CED-1::GFP (yellow arrows). (**G**) Quantification of apoptotic cells per gonad of wild-type (n=7), *his-59(kog7)* single mutant (n=5), and *his-59(kog7); his-55(kog8)* double mutant (n=7). All quantifications = average ± SE; *P*-value: unpaired t test, ** $P<0.01$, * $P \leq 0.05$, ns: not significant. Scale bars:5 μm in (**A, C** and **F**).

**Supplemental Tables:**

**Table S1. Summary of histone H3-like, and histone H3.3-like expression in *C. elegans* hermaphrodites.**

| Gene (Chromosome) | Germ -line | Sperm | Oocyte | Pre-gastrulation | Gastrulation | Source |
|---|---|---|---|---|---|---|
| *his-45(H3) (IV)* | + | + | + | + | + | This study |
| *his-59(H3) (IV)* | + | + | + | + | + | This study |
| *his-63(H3) (IV)* | + | + | + | + | + | This study |
| *his-55(H3) (IV)* | + | + | + | + | + | This study |
| *his-2(H3) (V/HIS1)* | - | - | - | - | + | This study |
| *his-6(H3) (V/HIS2)* | - | - | - | - | + | This study |
| *his-9(H3) (II/HIS3)* | - | - | - | - | + | This study |
| *his-13(H3) (II/HIS3)* | - | - | - | - | + | This study |
| *his-17(H3) (V/HIS4)* | - | - | - | - | + | This study |
| *his-25(H3) (II/HIS3)* | - | - | - | - | + | This study |
| *his-27(H3) (V/HIS4)* | - | - | - | - | + | This study |
| *his-32(H3) (IV/HIS5)* | - | - | - | - | + | This study |
| *his-42(H3) (II/HIS3)* | - | - | - | - | + | This study |
| *his-49(H3) (V)* | - | - | - | - | + | This study |
| *his-40(H3) (X)* | - | - | - | - | + | This study |
| *his-72(H3.3) (III)* | + | + | + | + | + | This study & Delaney et al. (ref 35) |
| *his-71(H3.3) (X)* | + | n/a | + | + | + | This study & Delaney et al. (ref 35) |
| *his-69(H3.3-like) (III)* | - | - | - | - | - | Delaney et al. (ref 35) |
| *his-70(H3.3-like) (III)* | + | + | - | - | - | Delaney et al. (ref 35) |
| *his-74(H3.3-like) (V)* | + | + | + | + | PGC-restricted | Delaney et al. (ref 35) |

**Table S2.** ***C. elegans*** **strains used in this study.**

| Strain name | Genotype | Source | Comments |
|---|---|---|---|
| JHU42 | *his-45(kog16[his-45::Dendra2]) IV* | This study | Dendra2 fusion |
| JHU5 | *his-6(kog3[his-6::Dendra2]) V* | This study | Dendra2 fusion |
| JHU4 | *his-72(kog2[his-72::Dendra2]) III* | This study | Dendra2 fusion |
| JHU20 | *his-72(kog5[his-72::mCherry]) III* | This study | mCherry fusion |
| JHU19 | *his-55 (kog11[his-55::TEV::eGFP::3xFlag]) IV* | This study | eGFP fusion |
| JHU14 | *his-59 (kog7) IV* | This study | H3 homologue deletion |
| JHU15 | *his-59 (kog7) IV; his-55 (kog8) IV* | This study | H3 homologue deletion |
| JHU16 | *his-59 (kog7) IV; his-55 (kog9) IV* | This study | H3 homologue deletion |
| JHU18 | *his-6 (kog10) V* | This study | Histone H3 dominant negative *his-6(H113D)* |
| OH14454 | otIs587 [gcy-5(fosmid::SL2::NLS::GFP + ttx3p::mCherry]. otIs304 [hsp16-2p::che-1::3xHA::BLRP + rol-6(su10060] | CGC | GFP cell fate reporter |
| JHU45 | *his-6 (kog10) V;* otIs587 [gcy-5(fosmid::SL2::NLS::GFP + ttx3p::mCherry]. otIs304 [hsp16-2p::che-1::3xHA::BLRP + rol-6(su10060] | This study | GFP cell fate reporter with histone H3 dominant negative allele |
| GC1413 | *Rrf-1(pk1417) I; naSi2(mex-5p::H2B::mCherry::nos-2 3'UTR) II; teIs113(pie-1p::GFP::H2B::zif-1 3'UTR) V* | Jane Hubbard lab (Roy D., et al. (ref 49) | Germline reporter |
| MD701 | *bcIs39 [lim-7p::ced-1::GFP + lin-15(+)]* | Zhou et al. (ref 50) | Apoptotic germ cell reporter |
| JHU57 | *his-55(kog17[his-55::Dendra2]) IV* | This study | Dendra2 fusion |
| JHU59 | *his-63(kog18[his-63::Dendra2]) IV* | This study | Dendra2 fusion |
| JHU53 | *his-59(kog19[his-59::Dendra2]) IV* | This study | Dendra2 fusion |
| JHU39 | *his-2(kog20[his-2::Dendra2]) V* | This study | Dendra2 fusion |
| JHU40 | *his-25(kog21[his-25::Dendra2]) II* | This study | Dendra2 fusion |
| JHU37 | *his-13(kog23[his-13::Dendra2]) II* | This study | Dendra2 fusion |
| JHU41 | *his-32(kog22[his-32::Dendra2]) IV* | This study | Dendra2 fusion |
| JHU80 | *H3::Dendra2 in the HIS4 cluster (his-17, his-27, and/or his-49 ) V ** | This study | Dendra2 fusion |
| PHX2995 | *his-40(syb2995[his-40::Dendra2]) X* | This study | Dendra2 fusion |

| | | | |
|---|---|---|---|
| FAS46 | *his-72(uge30[gfp::his-72]) III* | Delaney et al. (ref 35) | GFP fusion |
| FAS84 | *his-71(uge45[gfp::his-71]) X* | Delaney et al., (ref 35) | GFP fusion |
| JHU106 | *his-55(syb3144[his-55::Ollas]) IV ; his-72(kog5[his-72::mCherry]) III* | This study | Ollas and mCherry fusions |
| JHU79 | *ncIs13[AJM-1::GFP]; his-55 (kog11[his-55::TEV::eGFP::3xFlag]) ; his-72(kog5[his-72::mCherry]); his-6 (kog10)* | This study | AJM-1::GFP, H3::GFP, H3.3::mCherry |
| ST65 | *ncIs13[AJM-1::GFP]* | CGC | GFP fusion |

*CRISPR/Cas9 Dendra2 knock-in reagents were designed to specifically edit a histone *H3* gene within the HIS4 histone cluster on chromosome V (see Figure S1A), which contains three histone *H3* genes *his-17*, *his-27*, and *his-49*. The genotyping results indicated that one or more of these three *H3* genes contains the *Dendra2* sequences, but their genomic DNA sequences are too similar to distinguish which one of the three contains the *Dendra2* sequence. Therefore, we have used this strain as an H3 reporter representing the entire HIS4 cluster activity.

**Table S3. Reagents used for generating CRISPR/Cas9 mediated fusion proteins, deletion strains, and point-mutations in *C. elegans*.**

| Allele | sgRNA target sequences (PAM sites in bold) | Repair template homology arms** or repair template (deletion and point-mutations) |
|---|---|---|
| *his-45(kog16[his-45::Dendra2]) IV* | GCGCGCTTAAATACCTTTT**TGG**<br>GCTTGCTCAACTACCAAAAA**AGG** | 5’gctaagcgagtcaccatcatgccaaaggata Tccaattggccagacgcatccgaggagagc gTgctcagcacgtgatgaacaccccgg gaattaacc<br>5’ ggccctaaagagggccgttgggttcggttaagtttt gagattaagcttActTaactaTcaaaaaaAgtatTtt accacacctggctgggcaggg |
| *his-6(kog3[his-6::Dendra2]) V* | GGTGGGGGTTTGAATCGAAA**CGG**<br>ATCGAAACGGTCTCAAACTC**TGG** | 5’cgccaagcgagtcaccatcatgccaaaggacatcc aattggccagacgtatccgaggagaacgtgctcagca cgtgatgaacaccccgggaattaacc<br>5’ctaaagagggccgttgggttcggtgAgAgtttg aatTgaaacAgtTtcaaaTtctAgaaatcagaaa tttaccacacctggctgggcagg |
| *his-72(kog2[his-72::Dendra2]) III* | AGTGCTTCGAGAATTCCTGA**TGG**<br>GAGCTTAAGCACGTTCTCCG**CGG** | 5’ccacgccaagcgcgtcaccatcatgccaaaggacat gcaactcgccagacgcatTcgTggagaGcgtgctca gcacgtgatgaacaccccgggaattaacc<br>5’ggaaaaatacgaggattatggtacaagttggattaaat gaatattaaaagtgcttTgagaattAgtAatgAagcttac cacacctggctgggcaggg |
| *his-72(kog5[his-72::mCherry]) III* | AGTGCTTCGAGAATTCCTGA**TGG**<br>GAGCTTAAGCACGTTCTCCG**CGG** | 5’ccacgccaagcgcgtcaccatcatgccaaaggacatg caactcgccagacgcatTcgTggagaGcgtgctcag cacgtgatggtgagcaagggcgaggag<br>5’ggaaaaatacgaggattatggtacaagttggattaaatg aatattaaaagtgcttTgagaattAgtAatgAagcttactt gtacagctcgtccatg |
| *his-55(kog11[his-55::TEV::eGFP::3xFlag]) IV* | CAATTGGCCAGACGCATCCG**AGG**<br>GCTTGCTCAACTACCAAAAA**AGG** | 5’ gcgagtcaccatcatgccaaaggatatccaattggccag GcgTatTcgGggagagcgcgctgagaacctctacttcca Aggag<br>5’gtggccctaaagagggccgttgggttcggttaagttttgag attaagcttgctTaactaTcaaGaaagAtatttacttgtcatc gtcatccttgtaatc |
| *his-55(kog17[his-55::Dendra2]) IV* | CAATTGGCCAGACGCATCCG**AGG**<br>GCTTGCTCAACTACCAAAAA**AGG** | 5’gcgagtcaccatcatgccaaaggatatccaattggccagGc gTatTcgGggagagcgcgctcagcacgtgatgaacac cccgggaattaac<br>5’gtggccctaaagagggccgttgggttcggttaagttttgaga ttaagcttgctTaactaTcaaGaaagAtatttaccacacctgg ctgggcag |
| *his-63(kog18[his-63::Dendra2]) IV* | GCGCGCTTAAATACCTTTTT**TGG**<br>GCTTGCTCAACTACCAAAAA**AGG** | 5’cgctaagcgagtcaccatcatgccaaaggatatccaattgg ccagacgtatccgaggagagcgtgctcagcacgtgatgaaca ccccgggaattaacc<br>5’ggccctaaagagggccgttgggttcggttaagttttgagatta agcttActTaactaTcaaaaaaAgtatttaccacacctggctg ggcaggg |
| *his-59(kog19[his-59::Dendra2]) IV* | GAGCGCGCTTAAATACCTTA**TGG**<br>AAGCTTACTTAACTACCATA**AGG** | 5’agtcaccattatgccaaaggatatccagctggccagac gtatccgaggagagcgcgctcagcacgtgatgaacaccc cgggaattaacctg<br>5’ggccctaaagagggccgttgggttcggtgagttttgagtt |

| | | |
|---|---|---|
| | | gaagcttacttaaTtaTTataagAtatttaccacacctggct gggcaggggg |
| *his-2(kog20[his-2::Dendra2]) V* | CGGTGGGGTTTGAATTGAAA**CGG** AAATTTAAGCACGTTCTCCT**CGG** | 5'gccaagcgagtcaccatcatgccaaaggacatccaattg gccagacgtatTcgCggagaGcgtgctcagcacgtgat gaacaccccgggaattaacc 5'ccctaaagagggccgttgggttcggtgAAgtttgaattA aaacgAtctcaaactttctgaaaatcagaaatttaccacacct ggctgggcagg |
| *his-25(kog21[his-25::Dendra2]) II* | GCTGGCTCAGTACCATTGGA**AGG** TCAAGCTGGCTCAGTACCAT**TGG** | 5'ctaagcgagttaccattatgccaaaggacatccaattggca Agacgtatccgaggagagcgtgctcagcacgtgatgaacac cccgggaattaacc 5'gtggccctaaagagggccgttgggttcggttagattttgag atcaagctgActTagtaTcattAgaagAcatTTAccaca cctggctgggcaggg |
| *his-32(kog22[his-32::Dendra2]) IV* | AGCGTGCTTAAATGTCTTTG**TGG** ACATTTAAGCACGCTCTCCT**CGG** | 5'cacgctaagcgagttaccatcatgccaaaggatatccagct ggccagacgcatTcgaggagaAcgtgctcagcacgtgatg aacaccccgggaattaacc 5'gtggccctaaagagggccgttgggttcggttatttgagatca agcttgtacaaaatatcTacaaagaTatttaccacacctggctg ggcagg |
| *his-13(kog23[his-13::Dendra2]) II* | GCTGGCTCAGTACCATTGGA**AGG** TCAAGCTGGCTCAGTACCAT**TGG** | 5'ctaagcgagttaccattatgccaaaggacatccaattggcaa Gacgtatccgaggagagcgtgctcagcacgtgatgaacaccc Cgggaattaacc 5'gtggccctaaagagggccgttgggttcggttagattttgaga tcaagctAgTtcagtaTcattAgaagAcatTTAccacac ctggctgggcaggg |
| *H3::Dendra2 in the HIS4 cluster (his-17, his-27, and/or his-49) V ** | ACTCTGAAAATCAGAAATTT**AGG** AAATTTAGGCACGTTCTCCT**CGG** | 5'cacgccaagcgagtcaccatcatgccaaaggacatccaatt Ggccagacgtattcggggagagcgcgctcagcacgtgatgaa Caccccgggaattaac 5'gccctaaagagggccgttgggttcggtgggggtttgaatcga aacggtctcaaactctgaaaatTaAaGatttaccacacctggct gggcagg |
| *his-59 (kog7) IV* | CCCACGGATTATCAACCTAA**AGG** GAGCGCGCTTAAATACCTTA**TGG** | 5'ggcagccgttagtttcacttttctcacagtcccccaTA gattatcaaTctaaagAcaGCTACGataTcttatA gtagttaagtaagcttcaactcaaaactcaccgaacccaa cggccctc |
| *his-55 (kog9) IV* | GATTATCAACCTAAACGCAA**TGG** GCGCGCTTAAATACCTTTTT**TGG** | 5'ggcagccgttagtttcacttttctcacagtcccccaTA gattatcaaTctaaagAcaCCGGACTGTTAGT TGTTCAAAGGataTcttatAgtagttaagtaagct tcaactcaaaactcaccgaacccaacggccctc |
| *his-6 (kog10) V* | GGTGGGGGTTTGAATCGAAA**CGG** | 5'gtcggactcttcgaggacaccaacttgtgcgcaatcGAC gccaagcgagtcaccatcatgccaaaggacatccaattgg ccagacgtatccgaggagaacgtgcttaaatttctgatttcT agaAtttGATATCgtttcAattcaaacTcTcaccgaa cccaacggccctc |

*CRISPR/Cas9 Dendra2 knock-in reagents were designed to specifically edit a histone *H3* gene within the HIS4 histone cluster on chromosome V (see Figure S1A), which contains three histone *H3* genes *his-17*, *his-27*, and *his-49*. The genotyping results indicated that one or more of these three *H3* genes contains the *Dendra2* sequences, but their genomic DNA sequences are too

similar to distinguish which one of the three contains the *Dendra2* sequence. Therefore, we have used this strain as an H3 reporter representing the entire HIS4 cluster activity.

** for fluorescent protein tagging, Dendra2, mCherry, and eGFP were inserted into the endogenous locus to generate either H3 or H3.3 fusion proteins using a *dpy-10* co-CRISPR strategy *(30)*. Custom crRNA sequences were designed to target the sgRNA target sequence listed. High-fidelity PCR was used to generate a linear repair template with 35 bp homology arms. Sanger sequencing was used to confirm the accuracy of the knock-in allele.

**Supplemental Movie 1. H3.3 (HIS-72::mCherry) and H3 (HIS-55::Ollas) with H3K36me2.** 3D immunofluorescence images of HIS-72::mCherry (red), HIS-55::Ollas (green), and H3K36me2 (magenta) in pachytene nuclei were acquired using Airyscan microscopy. Movie rotates 360 degrees while alternating red, green, and magenta channels to visualize enrichment of H3.3, H3, and H3K36me2, respectively.

**Supplemental Movie 2. H3 (HIS-55::Ollas) and H3.3 (HIS-72::mCherry) with H3K27me2/3.** 3D immunofluorescence images of HIS-72::mCherry (red), HIS-55::Ollas (green), and H3K27me2/3 (magenta) in pachytene nuclei were acquired using Airyscan microscopy. Movie rotates 360 degrees while alternating red, green, and magenta channels to visualize enrichment of H3.3, H3, and H3K27me2/3, respectively.

**Supplemental Movie 3. Time-lapse movie of an embryo expressing H3.3 (HIS-72::mCherry) and a Class I H3 (HIS-55::GFP).** Live cell imaging of H3.3 (HIS-72::mCherry) and Class I H3 (HIS-55::GFP) during early embryogenesis. The dashed circles outline the P-lineage through multiple cell divisions. The video begins one frame prior to the first embryonic cell division and captures early embryogenesis until the P-lineage divides to generate the Z2/Z3 primordial germ cells. The video was acquired at 5-minute intervals. Snapshots are shown in Figure 2C.
